# New evidence on learning trajectories in a low-income setting

**DOI:** 10.1016/j.ijedudev.2021.102430

**Published:** 2021-07

**Authors:** Natalie Bau, Jishnu Das, Andres Yi Chang

**Affiliations:** a UCLA, United States; b Georgetown University, United States; c World Bank, United States^1^

**Keywords:** Test scores, Learning profiles, Dropouts, Teaching at right level, Primary schools

## Abstract

Using a unique longitudinal dataset collected from primary school students in Pakistan, we document four new facts about learning in low-income countries. First, children’s test scores increase by 1.19 SD between Grades 3 and 6. Second, going to school is associated with greater learning. Children who dropout have the same test score gains prior to dropping out as those who do not but experience no improvements after dropping out. Third, there is significant variation in test score gains across students, but test scores converge over the primary schooling years. Students with initially low test scores gain more than those with initially high scores, even after accounting for mean reversion. Fourth, conditional on past test scores, household characteristics explain little of the variation in learning. In order to reconcile our findings with the literature, we introduce the concept of “fragile learning,” where progression may be followed by stagnation or reversals. We discuss the implications of these results for several ongoing debates in the literature on education from Low- and Middle-Income Countries (LMICs).

## Highlights

• This paper uses unique longitudinal data to study learning trajectories through primary school in Pakistan.·
• Children’s test scores increase by 1.19 standard deviations between Grades 3 and 6, commensurate with gains observed in other countries.
• These gains reflect learning due to schooling rather than aging: children who dropout from school learn significantly less than those who remain.
• There is significant variation in how much children learn through primary school: those with the lowest test scores experience the largest gains.
• We introduce the new concept of `fragile learning’, showing that progression is frequently followed by stagnation or reversals in the data.

## 1 Introduction

What children learn during primary school and how learning varies by sex, parental background, and initial test scores is of critical importance for education policy. Moreover, the process of learning and the variation across subgroups may be very different in Low-Income Countries (LICs) from High-Income Countries. For instance, there is suggestive evidence that children with initially lower test scores learn less in LICs due to “curricular mismatch” because curricular standards are set substantially higher than children’s level of preparation (Pritchett and Beatty, 2015; Banerjee et al., 2016 and 2017, Kaffenberger and Pritchett, 2020a; Muralidharan et al., 2019). Despite a shift in emphasis in the literature on education in LICs from enrollment to learning and a large body of evaluative research on how to improve test scores, there is currently little data on learning trajectories through primary school. We address this vacuum, which is driven by the lack of large, longitudinal datasets of children’s test scores during their primary schooling years, in this paper.

We use a rich longitudinal dataset of children’s test scores over four years of primary schooling in Pakistan, collected through the Learning and Educational Achievement in Punjab or LEAPS project, to document four new facts about learning in low-income countries. First, we quantify how much children learn in school. We find that an aggregate (mean) test-score measure of learning increased by 1.19 SD between Grades 3 and 6. This implies that a high-performing child at the 75th percentile of the Grade 3 distribution “knew” as much as a low-performing child at the 25th percentile by Grade 6. Data from two comparison groups – the Young Lives surveys of Peru, Vietnam, India, and Ethiopia, as well as administrative data from Florida^2^ – show that, in all these settings, the rate of learning is similar (approximately 1 SD over 4 years). Thus, gains across years, *when measured relative to cross-sectional variation*, are similar in a highly disparate sample of countries.

Second, this learning does not solely reflect natural gains in reading, writing, and arithmetic skills as children age. To distinguish “learning due to aging” from “learning due to schooling,” we tracked and tested children who had dropped-out between Grades 5 and 6, a transition that requires a change from primary to middle school and may require more travel.^3^ In our data, the children who remained in school between these grades gained 0.40 SD, while there was no statistically significant increase in test scores among dropouts. This could be because dropouts were negatively selected—perhaps they dropped-out because they were not learning. However, while dropouts reported slightly lower (but not statistically significantly lower) test scores than children who continued in school, learning *gains* between Grades 3 and 5 were identical for dropouts compared to those who remained in school. This is consistent with the ‘parallel trends’ assumption required for this type of difference-in-difference estimate and suggests that the gains we observe can be causally attributed to schooling itself.

Third, we find significant variation in how much children learn in our sample. The bottom decile of “learners” in terms of test scores gains (defined as the difference between final and initial test scores) *lost* 0.49 SD. The second decile gained 0.39 SD, and the top decile gained 2.77 SD over the four years of data. The strongest determinant of how much a child learns between Grades 3 and 6 is her initial test score. Children whose test scores were in the bottom 20 % in Grade 3 learn significantly more, gaining 1.75 SD between Grades 3 and 6, than children ranked in the top 20 % (0.71 SD). Accounting for measurement error reduces the relative gains of the lowest performers but does not reverse the pattern.

Fourth, conditional on past test scores, household characteristics explain little of the variation in test scores between Grades 3 and 6. Regression estimates suggest that 56 % of the variance in test score levels in a given year is explained by lagged test scores, but including a full set of village and school fixed effects, as well as parental and child characteristics, explains only another 6% of the variation. We focus on two characteristics—gender and family wealth—in greater detail and show that our results are robust to alternate methods of test scaling, an issue that has received considerable attention in recent work from the United States.

These findings significantly broaden what we know about learning in low-income countries. Our first finding – that students’ test scores increase by more than 1 SD on average over the course of 4 years of primary school – contrasts with several studies arguing that many children progress very slowly through schools with learning trajectories that “flatten” over the years (Beatty et al., 2018; Filmer et al., 2006; Kaffenberger and Pritchett, 2020b; Pritchett and Sandefur, 2020). Yet, there are few school panels with calibrated test questions that can be used to answer this basic question. In our novel panel, schooling *does* appear to be associated with learning, at least on average. While comparing the magnitude of learning gains measured with different tests across countries is difficult, we cannot reject that learning gains are similar across a variety of settings.

Our second finding that students who attend school experience test score gains while dropouts do not contrasts with past studies that have shown that programs incentivized to retain children in school and increase enrollment do not improve test scores among treated cohorts (Hanushek et al., 2008; Behrman et al., 2009; Filmer and Schady, 2019; Zuilkowski et al., 2016; Nakajima et al., 2018).^4^ Importantly, our results do not imply that test scores and dropout are not correlated. Even for the limited sample of children moving between Grades 5 and 6, this correlation is positive.^5^ However, we are not aware of studies that have tracked children for multiple years and compared the test-score trajectories of children who dropout versus those who choose to continue. The specific result that there is no correlation between dropping out and test score *gains* is new to the literature.

Our third finding – that test score gains are highly variant and largest among initially low performers – relates to several studies that argue that many children, especially low performers, often start off learning very little and that their learning trajectories flatten as they fall behind and stop learning at some point during primary school (Beatty et al., 2018; Filmer et al., 2006; Kaffenberger and Pritchett, 2020b; Muralidharan et al., 2019). We find the opposite pattern in our data. We discuss how the disparity in findings across settings can be explained by subtleties in how learning is measured. Measuring learning in terms of test score gains, as we do, would lead researchers to conclude that learning is highest among the initially poorly-performing. Measures of learning that assume imperfect persistence of initial test score levels with a common persistence parameter (e.g. Muralidharan et al., 2019) lead to the opposite conclusion.

We further contribute to the literature by introducing the novel concept of “fragile learning” to reconcile these findings. In our setting, as in most settings, test scores are *not* highly persistent across years. One extreme consequence of low persistence is that learning trajectories are not monotonically increasing for all children or all questions. In fact, a sizeable fraction of children experience test score losses every year. Item-wise analysis shows that the fraction of children whose performance for specific questions features gains followed by losses is as high as the fraction of children who are “robust learners,” or children whose learning trajectories show either stability or monotonic increases every year. The difference between our results and those of Muralidharan et al. (2019) reflects how different specifications account for fragile learning — unconditional test score *levels* converge, with low performers increasing their test scores relatively more, but test score levels conditional on an imperfectly persistent baseline test score do not. Understanding the causes and pedagogic basis of low persistence in low-income countries is a fertile area for further investigation.

Our fourth finding – that wealth and gender explain little of the variation in current test scores conditional on lagged test scores – is surprising, especially given a long history, dating back to the Coleman report, of associating performance in tests with the home environment (Coleman, 1975). However, it accords with more recent results from high-income countries, where the difference between the correlates of levels and gains has become more apparent with better data. A number of studies now show that family background is strongly correlated with test score gains in the pre-school years but not necessarily associated with gains (*not* levels) during the primary schooling years (Reardon, 2011 and 2013).

The remainder of the paper is organized as follows. We first describe the LEAPS data in Section 2 and follow this with a description of the main patterns in Section 3. Section 3 also presents our regression estimates and discusses how we address attrition and measurement error in the sample. Section 4 introduces the concept of fragile learning, and Section 5 concludes with a brief discussion. We emphasize that this is a “first look” at the data, and there is considerable room for further research, both from an educational and psychometric perspective.

## 2 Context and data

### 2.1 Population and sampling

The LEAPS study was started in 2003 in the province of Punjab in Pakistan, which has an approximate population of 70 million people and is the 12th largest schooling system in the world. A sample of 112 villages was drawn from three districts —Attock in the North, Faisalabad in the center, and Rahim Yar Khan in the south— following an accepted stratification of the province along educational outcomes into the better performing center and north and the poorly performing south. The sample was drawn from villages with at least one private school, consistent with LEAP’s goal of understanding the role of private schooling. For each of these districts, the list frame consisted of all villages that had at least one private primary school within the relevant “choice set” for households in the village (schools in the village or schools within a 15 min boundary of the village in Attock and Faisalabad and a 30 min boundary in Rahim Yar Khan). In these villages, all schools in the choice-set were covered as part of the LEAPS project, resulting in a total of 823 public and private schools in 2003. Between 2003 and 2006, we carried out tests in 1121 schools in 119 villages.^6^ The higher number of schools (1121 versus 823) both reflects the exit and entry of schools and our sampling strategy where we attempted to track and follow children at whatever school they were in.

Andrabi et al. (2008) have compared the villages in the LEAPS sample to representative samples from Punjab and show that these villages tend to be larger and wealthier, with greater access to infrastructure. This follows from the restriction in the sample-frame that each village should have at least one private school. Therefore, the learning patterns that we present here are not representative of either remote rural villages or urban areas. Nevertheless, the range of household and school characteristics in the LEAPS sample covers most villages in Punjab except for the poorest, since 60 % of Punjab’s rural population lives in a village with access to at least one private school (Andrabi et al., 2008).

Andrabi et al. (2008) also characterizes the households and schools in these villages. Noteworthy features of the sample in 2003, when the first survey was collected are: (a) 50 % of household heads in the sample reported no education at all; (b) about a quarter of household heads reported their primary occupation as farming; and (c) the average household size was 7.5 members. Enrollment patterns reflect those in many low-income countries, with 76 % of boys between the ages of 5 and 15 enrolled in school in 2003 compared to 65 % of girls, and a classic inverted U-shape in enrollment-age profiles, reflecting both late entry into schooling and dropouts from age 11 onwards. Although we do not focus on learning differences between private and public schools here (see Andrabi et al., 2020 for a causal analysis of private schooling and test scores), we note that 70 % of enrolled children were in public and 28 % in private schools. Enrollment in religious schools or madrassas was only 1%, reflecting nationwide enrollment patterns (Andrabi et al., 2006).^7^

### 2.2 Data collection and samples

We use three datasets collected as part of the LEAPS surveys. These are (1) data on test scores, (2) data on family characteristics, and (3) data collected from households. The first two datasets were collected at schools and we refer to these samples as the “School Sample.” The sample in the third dataset, which was collected at households, is referred to as the “Household Sample.”

#### 2.2.1 Test scores in the school sample

In each year between 2003 and 2006, the LEAPS study tested children using tests designed in consultation with pedagogical and education experts in the subjects of English, Urdu, and Mathematics. Tests were administered by the LEAPS team and were then recovered at the end of the test, minimizing the possibility of manipulation or cheating. The norm-referenced tests covered a wide range of concepts and capabilities in order to track how children learned over time. See Andrabi et al. (2002) for a detailed description of the test. The tests were first administered in 2003 to children in Grade 3 and then again in 2004, 2005, and 2006, as the majority of the children transitioned to Grades 5 and 6.^8^ New children found in the appropriate grade each year were also tested at the school. Each test retained a rotating core of “linking items,” so that some questions were repeated from year to year. These linking items allow us to calibrate all the tests on a common scale, following established methods in the literature on Item Response Theory (Hambleton and Swaminathan, 2013).

Tracking children over time for test data was challenging, especially as children do not have methods of identification and switch schools over the multiple years of the survey. Every year, the LEAPS team conducted an extensive tracking exercise, where we tried to ascertain the whereabouts of each child enrolled in the relevant grade in the past year. Most children remained in the same school, but 5−7 % switched schools. Sending schools had no information on whether these children had dropped-out, left the village, or were enrolled elsewhere, and teams spent several weeks trying to track the location of these children.^9^ We will further discuss below how this affects the data.

#### 2.2.2 Family characteristics data for the school sample

On the day of the test in 2003, we sampled 10 children randomly from each class and completed a short questionnaire with these students on their family background. In subsequent years, we continued to complete the questionnaire with these children and additionally surveyed randomly selected children from the same classroom.

#### 2.2.3 Household sample

Our third data source comes from a concomitant household survey carried out among 1875 households in 2003 in the same villages. The household survey was designed to complement the school survey and oversampled households with children between the ages of 9 and 11. In cases where such a child was located in the household and was enrolled in the school, we have the test score of the child and parental background variables that have been collected by the surveyors from the parents themselves. Furthermore, in 2006, we were worried about extensive missing test scores as children transitioned from primary to middle school. Therefore, to retain one consistent panel, we also tested children who had been part of the school test-score panel during home visits. As we will discuss in more detail, the household sample allow us to (1) assess how sensitive our results are to attrition and (2) compare the learning trajectories of students who dropout and remain in school.

### 2.3 Measurement

In this subsection, we describe the two important components of our strategy to measure students’ learning. We first discuss the measurement of test scores with an item response model, and then describe how we translate these test scores into our key measure of learning throughout the paper.

#### 2.3.1 Test score measurement

In the Item Response Model, the likelihood of answering a question correctly is determined by the ability of the child, labelled θ, and item parameters, labelled a, b, and c for difficulty (a), discrimination (b), and a guessing parameter (c).^10^ If there are N children and M questions, then N + 3 M parameters are estimated through the IRT method, one θ for each child, and 3 parameters for each of the M questions. For each item, the estimation produces an “Item Characteristic Curve” that provides, for each θ, the likelihood that a question is answered correctly. The item characteristic curve is given by the 3-parameter logistic:

$$P_{j}(\theta )=c_{j}+(1-c_{j})\frac{1}{1+\text{e}\text{x}\text{p}\{-a_{j}\left(\theta -b_{j}\right)\}}$$

where $c_{j}$ is defined as the guessing parameter, since it’s the probability of getting the question right through pure guessing; $b_{j}\;\equiv \theta^{*}|P_{j}\left(\theta^{*}\right)=\frac{1+c_{j}}{2}$ and is the difficulty parameter, which is the ability level at which the child will answer the question correctly half the time (adjusted for guessing), and $a_{j}\propto \frac{\partial P_{j}(\theta )}{\partial \theta }$ at $\theta =b_{j}$, is the discrimination parameter, which specifies the steepness of the item characteristic curve at the point that the ability of the child is equal to the difficulty of the question ($b_{j})$.

The joint estimation of these parameters follows the standard procedure in IRT using the IRT command, *OpenIRT*, developed by Zajonc for STATA and discussed in Das and Zajonc (2010). In order to maximize efficiency, we use all the data available by pooling test score data from 2003 to 2006 to jointly estimate θ and the item parameters.^11^ An observation is at the child-year level, so test score gains are given by the difference in θ for any child across two (or more) years. Even though the test can change in each year, the exercise requires that across any two years, there are *some* common questions that can be used to “link” items. Item parameters for these questions are assumed to be time invariant, allowing us to identify parameters for other questions that are not common across years and place every θ on a common scale. We describe how we assess item-invariance below. As θ can only be identified up to an arbitrary scale and origin, we follow the convention that θ is drawn from a distribution with mean 0 and SD (approximately) equal to 1 across the entirety of the sample.^12^

#### 2.3.2 Measuring learning

With the test score measures in hand, we identify the key object of interest in this paper – learning gains. Our measure of a student’ s learning gain from period $t_{0}$ to period T, which we also refer to as the student’s *test score trajectory*, is given by $y_{iT}-y_{{i,t}_{0}}$. In the literature (see, for example, Muralidharan et al., 2019), annual learning is sometimes alternatively measured with a value-added specification $y_{iT}-\beta y_{i,T-1}.$ In this specification, β, identified from the data, captures the imperfect persistence of test scores over time resulting from both measurement error and forgetting. For much of this paper, we focus on the test score trajectory specification, since it exactly captures how much a student’s level of knowledge (as proxied by her test score) increased over time. In Section 4, in our discussion of fragile learning, we further compare these measures and point out areas where these measures can lead to different conclusions.

### 2.4 Threats to the validity of measures

#### 2.4.1 Validation of the IRT model in the LEAPs data

The IRT model’s linking procedure assumes that test parameters are invariant over time so that the increased likelihood of answering a particular question in a particular year is fully determined by a change in the child’s θ. This is an assumption about the stability of the item characteristic curve and assumes that, as children progress, they move along the estimated curve, while the curve itself does not change. It is similar to the assumption of “no differential item functioning” for horizontal test equating—for any two groups (say, by race), the likelihood of answering a question correctly should depend only on underlying ability and not group membership.^13^

**Fig. A1:**
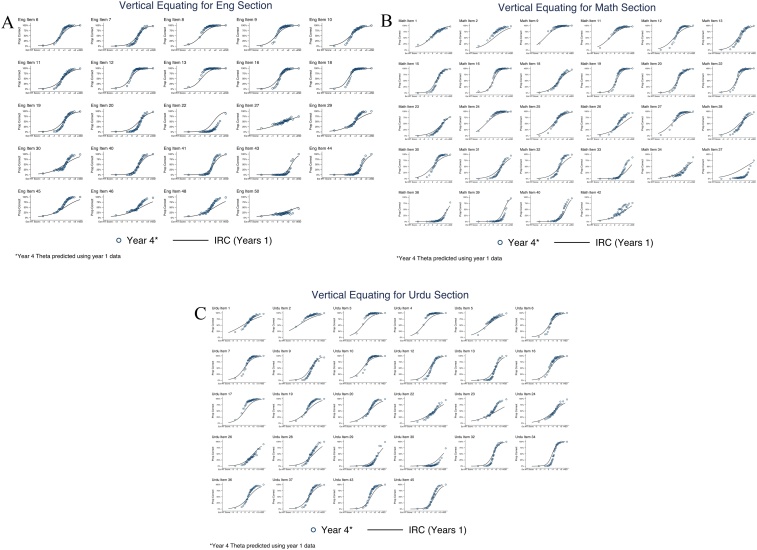
Vertical equating by subject. Notes: This figure shows the results of a vertical equating exercise by subject. First, item parameters from year 1 only are estimated. Then, the item parameters are assumed to be fixed and used to re-estimate new θ’s for children using their patterns of responses for common items in year 4. The solid line in each graph is the item characteristic/response curve, which represents the expected patterns of responses for each θ. The actual patterns of responses against θ for 40 quantiles is then plotted against it. If the expected and actual patterns of responses match, this implies that children are moving along a fixed item characteristic curve and that the curve itself is not shifting across years. For 9/24 English questions, the Pearson’s Chi^2^ test of differences is significant between the observed and expected frequencies of answering correctly when dividing the sample in 1000 quantiles by subject theta for a total sample of 10,067 or about 10 students by quantile. For 6/28 Math questions, the Pearson’s Chi^2^ test of differences is significant between the observed and expected frequencies of answering correctly when dividing the sample in 1000 quantiles by subject theta for total sample of 10,067 or about 10 students by quantile. For 4/28 Urdu questions, the Pearson’s Chi^2^ test of differences is significant between the observed and expected frequencies of answering correctly when dividing the sample in 1000 quantiles by subject theta for total sample of 10,067 or about 10 students by quantile.

This is a strong assumption but one that can be empirically tested. Appendix Fig. A1 shows the results for an exercise where we first estimate item parameters from year 1 (2003) only, along with the distribution of θ for the children in that sample. We then assume that the item parameters are fixed and using the same parameters, we re-estimate new the new distribution of θ using their patterns of responses for common items in year 4 (2006). We then plot (solid line) the expected patterns of responses for each θ (the “item characteristic curve”) and the actual patterns of responses against θ. If the expected and actual patterns of responses match, this implies that children are moving along a fixed item characteristic curve and that the curve itself is not shifting across years.

For most items, we find a close match between the expected and observed response patterns. Many items match almost exactly suggesting that vertical linking is possible in this setting and for this test, but there are also some notable departures. Observed response patterns for Urdu Item 23, for instance, are far above the expected response patterns with fixed item parameters at θ above the mean. This particular question asks children to select the correct antonym for the word “victory” among the options (a) “success,” (b) “defeat,” and (c) “weapon.” Another example is English Item 22 (which asks children to fill in the missing letters to complete the word “fruit” next to a picture of fruits). Here, the observed patterns are worse than expected suggesting that similarly knowledgeable children find it harder to answer this question correctly in higher grades. It could be, for instance, that the Grade 3 curriculum included the word “fruit,” but the Grade 5 curriculum did not have this particular word.

We tested these departures using Chi-Squared tests and are unable to reject equality for 61 of 80 common items across years 1 and 4. We have also recomputed test score gains after (a) dropping the questions where vertical equating seems to fail; and (b) including all questions, but fixing item parameters for the 61 items where they appear to be invariant and leaving item parameters to be estimated for the other 19, where vertical equating seems to fail (see Appendix Table A1). We observe no appreciable difference in the patterns of test score gains. Based on these exercises, we are cautiously optimistic that vertical linking is viable with these data. We emphasize however, that this is an area that requires further refinement and investigation for this dataset.

#### 2.4.2 Attrition

Attrition is a common problem in the collection of school-based test score data in low-income countries, where student absenteeism rates can range from 10 % to 20 % on any given day due to sickness or other emergencies at home. In our dataset, there are 16,428 unique children who appear 47,105 times over the 4 years. Of these unique child-year observations, 51 % correspond to observations in every year, and 82 % to observations in at least 3 years. To address attrition, we pursue two avenues. Appendix A explores the underlying dynamics that could produce similar attrition patterns to those we observe in Table 2. We conclude that attrition is primarily due to a combination of random absence and a small degree of miscoding in child IDs; however, we cannot rule out some degree of selection as well. Therefore, in Section 3, we will discuss the potential bias induced through attrition by comparing samples who are more and less intensively tracked and conclude that it is small.

## 3 Results

Our main results section proceeds in four parts, with each part describing one of the new facts generated by the LEAPS data. Many of our results can be presented through tables and figures of means, and this is what we focus on, augmenting these results with regression estimates to provide standard errors and show robustness to attrition, measurement error, and test-score scaling.

### 3.1 How much do children learn?

Using the unbalanced school sample, Table 1 shows what children learn during primary schooling, focusing on specific items that were repeated in every year. When first tested in Grade 3, most children could match simple words in English to pictures (such as “book”), add and subtract 2-digit numbers, and recognize Urdu alphabets and how to combine them into simple words (Urdu 10). But they could not construct simple sentences in English (such as “I play” or “The water is deep”), multiply or divide, or read an Urdu passage.

**Table 1:** Proportion of correct answers by subject for anchoring items across grades 3-6.

	Round 1 Grade 3 2003	Round 2 Grade 4 2004	Round 3 Grade 5 2005	Round 4 Grade 6 2006
*Number of Children*	12,109	12,806	12,123	10,067
English				
Eng 6: Listen to word, write word (boy)	0.39	0.52	0.65	0.74
Eng 7: Listen to word, write word (girl)	0.20	0.24	0.32	0.46
Eng 8: Alphabet order, fill in blank letter (e)	0.70	0.78	0.88	0.90
Eng 9: Alphabet order, fill in blank letter (m)	0.59	0.67	0.79	0.82
Eng 10: Alphabet order, fill in blank letter (s,t)	0.50	0.58	0.69	0.71
Eng 11: Alphabet order, fill in blank letter (n)	0.32	0.41	0.54	0.60
Eng 12: Match picture with word (banana)	0.61	0.71	0.82	0.85
Eng 13: Match picture with word (book)	0.70	0.80	0.89	0.93
Eng 16: Fill missing letter for picture (ball)	0.45	0.49	0.64	0.71
Eng 18: Fill missing letter for picture (cat)	0.67	0.71	0.80	0.83
Eng 19: Fill missing letter for picture (flag)	0.28	0.28	0.46	0.53
Eng 20: Fill in blank letters of word w/ picture (elephant)	0.17	0.17	0.25	0.34
Eng 22: Fill in blank letters of word w/ picture (fruit)	0.09	0.07	0.10	0.11
Eng 27: Check antonym of word (rough)	0.29	0.34	0.41	0.49
Eng 29: Fill missing word in sentence (his)	0.30	0.34	0.51	0.61
Eng 30: Fill missing word in sentence (show)	0.27	0.32	0.43	0.51
Eng 40: Construct sentence with word (school)	0.11	0.15	0.29	0.44
Eng 41: Construct sentence with word (doctor)	0.07	0.09	0.21	0.37
Eng 43: Construct sentence with word (deep)	0.01	0.01	0.03	0.10
Eng 44: Construct sentence with word (play)	0.02	0.03	0.10	0.20
Eng 45: Read passage and answer questions	0.27	0.35	0.52	0.67
Eng 46: Read passage and answer questions	0.21	0.30	0.40	0.53
Eng 48: Read passage and answer questions	0.17	0.24	0.39	0.51
Eng 50: Read passage and answer questions	0.10	0.14	0.18	0.21

Math				

Math 1: Count and write number (8)	0.60	0.65	0.78	0.73
Math 2: Count and check number (2)	0.46	0.51	0.69	0.78
Math 9: Add, subtract (3 + 4)	0.89	0.90	0.94	0.93
Math 11: Add, subtract (9 + 9+9)	0.74	0.79	0.86	0.86
Math 12: Multiply (4 × 5)	0.58	0.60	0.73	0.79
Math 13: Fill in blank multiply (2x_ = 20)	0.38	0.42	0.52	0.61
Math 15: Write word from number (113)	0.26	0.27	0.47	0.55
Math 16: Write number for word (18)	0.51	0.62	0.79	0.84
Math 18: Read and write time (12 -h clock showing 3:40)	0.24	0.28	0.47	0.53
Math 19: Word problem, find information and use	0.39	0.47	0.66	0.75
Math 20: Word problem, find information and use	0.35	0.44	0.59	0.67
Math 22: Word problem, find information and use	0.47	0.58	0.74	0.79
Math 23: Word problem, find information and use	0.09	0.12	0.20	0.29
Math 24: Add and subtract advanced (36 + 61)	0.84	0.86	0.91	0.92
Math 25: Add and subtract advanced (678 + 923)	0.54	0.56	0.69	0.72
Math 26: Add and subtract advanced (5.9 + 4.3)	0.20	0.35	0.55	0.58
Math 27: Add and subtract advanced (98−55)	0.69	0.73	0.81	0.84
Math 28: Add and subtract advanced (238−129)	0.32	0.38	0.48	0.51
Math 30: Multiply and divide (32 × 4)	0.50	0.53	0.68	0.73
Math 31: Multiply and divide (417 × 27)	0.13	0.15	0.30	0.36
Math 32: Multiply and divide (384/6)	0.19	0.23	0.43	0.51
Math 33: Multiply and divide (352/20)	0.01	0.02	0.16	0.23
Math 34: Cost of necklace, simple algebra	0.10	0.14	0.24	0.27
Math 37: Add and subtract fractions (1/2 + 3/2)	0.18	0.07	0.05	0.11
Math 38: Add and subtract fractions (7/5−3/4)	0.01	0.01	0.03	0.09
Math 39: Convert fractions and percentages (7/3)	0.02	0.04	0.06	0.13
Math 40: LCM (needed for adding with different denominator)	0.01	0.12	0.14	0.26
Math 42: Read scale and compare numbers	0.12	0.16	0.29	0.42

Urdu				

Urdu 1: Alphabet order, fill in blank letter (Cheeh)	0.57	0.61	0.68	0.70
Urdu 2: Alphabet order, fill in blank letter (Meem)	0.75	0.81	0.88	0.88
Urdu 3: Match picture with word (Kitaab)	0.71	0.78	0.90	0.93
Urdu 4: Match picture with word (Kaila)	0.71	0.78	0.89	0.93
Urdu 5: Match picture with word (Ghar)	0.52	0.57	0.67	0.74
Urdu 6: Dejoin letters of word into indiv letters (Mashraq)	0.46	0.56	0.69	0.75
Urdu 7: Dejoin letters of word into indiv letters (Sooraj)	0.56	0.65	0.77	0.81
Urdu 9: Dejoin letters of word into indiv letters (Abdul Majeed)	0.19	0.24	0.31	0.45
Urdu 10: Combine letters into joined word (Kaam)	0.72	0.76	0.85	0.88
Urdu 12: Combine letters into joined word (Maalik)	0.36	0.41	0.52	0.59
Urdu 13: Combine letters into joined word (Maheena)	0.09	0.14	0.24	0.35
Urdu 16: Check correct word to fill in sentence (Gehri)	0.41	0.49	0.67	0.76
Urdu 17: Check correct word to fill in sentence (Saaf)	0.53	0.65	0.82	0.87
Urdu 19: Antonyms (Bara)	0.42	0.47	0.65	0.77
Urdu 20: Antonyms (Geila)	0.35	0.45	0.60	0.67
Urdu 22: Antonyms (Buzdil)	0.22	0.27	0.35	0.45
Urdu 23: Antonyms (Shikushat)	0.20	0.24	0.42	0.54
Urdu 24: Antonyms (Mukhtasir)	0.24	0.28	0.40	0.48
Urdu 26: Write plurals of singular words (Aadat)	0.12	0.18	0.26	0.33
Urdu 28: Write plurals of singular words (Haraf)	0.13	0.23	0.37	0.48
Urdu 29: Write plurals of singular words (Sajar)	0.03	0.03	0.07	0.18
Urdu 30: Write plurals of singular words (Shaer)	0.01	0.02	0.03	0.06
Urdu 32: Construct a sentence with a given word (Karigar)	0.15	0.20	0.33	0.45
Urdu 34: Construct a sentence with a given word (Ghosila)	0.23	0.26	0.41	0.51
Urdu 36: Complete passage for grammar (Key)	0.28	0.35	0.53	0.65
Urdu 37: Complete passage for grammar (Chuka)	0.30	0.37	0.55	0.65
Urdu 43: Read passage and answer questions	0.21	0.32	0.56	0.66
Urdu 45: Read passage and answer questions	0.08	0.16	0.30	0.47

Notes: This table uses the full unbalanced sample and shows the proportion of correct answers for each item by subject and in each year (columns). Only anchoring items asked every year are included in the table. Questions left unanswered are marked as wrong and counted in the proportion. Note that while each year roughly corresponds to a primary grade, the sample tracks children who were observed in previous years even when they are not in their expected grade (e.g. children held back, double-promoted, etc.).

**Table 2:** Sample of children by number of years observed, child and household characteristics and mean learning.

N Years Observed	N Child-Year Obs.	% Obs.	N Unique Children	Female Proportion	Age (2003)	Avg Days Absent (last 30 days)	% Fathers w/ Primary Edu. or Less	% Mothers w/ Primary Edu. or Less	HH Assets PCA	Avg. Annual Learning
4	24,152	51.27	6038	0.48	9.58	1.82	44.24	76.13	0.11	0.39
3	14,280	30.32	4760	0.44	9.69	1.97	51.25	79.07	−0.01	0.40
2	6088	12.92	3044	0.38	9.90	2.10	48.50	78.32	−0.09	0.37
1	2585	5.49	2585	0.38	9.83	2.42	50.75	78.25	−0.48	–

Notes: This table uses the full unbalanced sample. The “number of years observed” categories are exclusive. Thus, children observed for 1 year are not counted again in other categories. Age in 2003 is estimated for those not observed in that year. Average annual learning is defined as the mean of learning between every year a child is observed. If there are 2- or 3-year gaps, then learning is divided by the number of years in the gap. Father and mother education groups (used to construct % of fathers and mothers with primary education or less) and household assets are not available for every child as these data was only collected for a subsample of children that have test scores. The household assets PCA is the average of all years observed, ignoring missing data. The household assets PCA index is very highly correlated (corr = .96) with an index constructed using IRT on the same household assets (see Appendix Fig. A5 for details on how these two measures compare). Fathers’ education, mothers’ education, and household asset information is from the school survey and was cleaned to make it stable across years (see Appendix Table A8 for details on how these variables were cleaned).

Tested again at the beginning of Grade 6, there are improvements in every item, typically in the range of a 15–30 percentage point greater likelihood of a correct answer. By this time, children were learning how to spell simple words in English, add and subtract larger numbers, and perform simple multiplication. Moreover, 51 % of children can divide 384 by 6, and a small minority can convert word challenges into Math or complete simple operations with fractions. For the vernacular, Urdu, it seems that learning has progressed sufficiently to allow students to fill in grammatically correct missing portions in a paragraph.

IRT scaling allows us to combine these item-level responses into a single score, which we report in Table 3. Here, the scores are computed for all students, and items across all four years and vertically equated using linking items as discussed previously. Table 3 shows that in Pakistan, between Grade 3 and the beginning of Grade 6 (ages 9.7–12.8), children have gained 1.08 SD in Mathematics and 1.29 SD in Urdu for a combined average increase of 1.19 SD across these two subjects.

**Table 3:** Learning between Grades 3-6, top vs. bottom 25 % in PK, YL countries and FL.

Country	Ages (t_0_ - t_1_)	Mean Score Difference t_1 –_ t_0_ (4 Years Learning)			75th Percentile at t_0_	25th Percentile at t_1_	Percentage in Correct Grade at t_1_
		*Math*	*Language*	*Combined*	*Combined*		
*Pakistan*	9.7−12.8	1.08	1.29	1.19	0.13	0.07	81% in Grade 6
*Florida*	9.2−12.2	1.04	0.99	0.99	0.01	−0.01	83% in Grade 6
*Ethiopia*	8.1−12.1	0.88	1.10	0.99	0.70	0.29	38% in Grades 4−6
*India*	8.0−12.0	0.98	1.17	1.08	0.04	0.57	54 % in Grades 5−7
*Peru*	8.0−11.9	1.12	1.42	1.27	0.72	1.08	32% in Grades 5−7
*Vietnam*	8.1−12.2	1.11	1.27	1.19	1.00	1.41	70 % in Grades 5−7

Sources: LEAPS, micro-data from the Young Lives (YL) Surveys provided by Abhijeet Singh, and analytical results using Florida administrative data facilitated by David Figlio.

Notes: This table shows the mean test score gains between t_1_ and t_0_ by subject and the 75th and 25th percentiles at t_0_ and t_1_ respectively for a range of countries/territories where panel data with equated test scores are available. For Pakistan and Florida, t_0_ = 2003 and t_1_ = 2006, for YL countries, t_0_ = 2009 and t_1_ = 2013. Language refers to receptive vocabulary for YL countries, reading for Florida, and Urdu for Pakistan. For YL countries, combined refers to the mean of Math and Language average scores as the sample of tested children did not always complete both subjects. For Pakistan and Florida, combined refers to the average score across Math and Urdu/reading, respectively. Pakistan and Florida are panels observed first at the school in Grade 3, while YL numbers come from household surveys where children are first tracked at age 5 and then followed at age 8 and 12. Children tested at home in Pakistan are excluded for comparability purposes with Florida. YL uses EAP IRT theta estimates standardized with respect to age 5 test scores. Attrition is low in all countries.

#### 3.1.1 Robustness to attrition

One important question is whether our estimated learning gains are biased due to attrition (or accretion). To evaluate the scope for attrition to affect our results, Table 2 reports the sample of children observed in each of the four years of testing in the school sample. We first show the number of rounds children appeared in, followed by basic child characteristics (sex and age), the average number of days absent in the last month (as reported by their teacher), family characteristics (parental education and assets), and their average annual test scores gains in the years they were observed. Although there are small differences in child and parental characteristics across samples, regardless of the sample of tested children we focus on (those observed for 2, 3, or 4 years), the average of our key metric of annual learning gains is approximately 0.39 SD. The stability of this value across rounds provides initial evidence that estimates of learning gains are not highly sensitive to selective attrition.^14^

We can further verify the extent to which test score gains in the schooling panel are unbiased by identifying a sample where the pattern of attrition is less severe. The intuition here is similar to the idea of “intensive tracking” or “double sampling” in clinical data (Baker et al., 1993). Specifically, if missingness is selective, the means computed from samples with ‘more’ and ‘fewer’ missing observations will be informative of the selection process. If children who are missing are highly selected, as missingness declines, we would expect to see meaningful changes in the estimated average test score gains. Appendix A presents a formal argument for this intuition.

Using the household sample, we are able to compare a more and less intensively tracked sample. We have constructed the analog to Table 2 for the household sample in Appendix Table A3. Here, the number of children on whom we have test scores is much smaller (1052), but 72 % of the unique child-year observations correspond to test scores observed in every year, and 92 % correspond to test scores observed in at least 3 of 4 years. Furthermore, test scores for students who are observed in all four years in the household and school sample track each other closely, with gains from the first to the fourth round of 1.13 SD for the school sample compared to 1.10 SD for the household sample (with yearly test score gains of approximately 0.38 SD).^15^ This suggests that selection bias is unlikely to strongly affect the average annual test gains estimates in our setting, as a substantial decline in attrition when we use the household instead of the school sample does not alter our main conclusions.

#### 3.1.2 How does learning in the LEAPS sample compare to other settings?

Our only recourse for comparison to other settings with similarly equated test scores is the Young Lives study, which tested children in Ethiopia, India, Peru and Vietnam, and data from Florida, where analysis was provided to us by David Figlio using administrative data from that state. This is far from an ideal comparison. LEAPS and Florida are school-level panels that tracked children who were first observed in Grade 3, while data in the Young Lives countries is collected at the household-level, and initial selection into the panel starts at age 8 rather than when children are enrolled in a specific grade. Furthermore, there are differences in the samples, with LEAPS testing children only in rural areas but in all schools and the Young Lives using a representative sample of urban and rural children in each of their settings.

Surprisingly, despite these substantial differences, test score gains over equivalent ages follow a very similar pattern, with increases of 1–1.27 SD in these other settings. The exceptions are language gains in Peru, which are notably higher, and Mathematics gains in Ethiopia, which fall below the average. Although the similarity in relative gains is striking, we stress that this tells us little about absolute learning across countries. Whether these patterns reflect more or less learning depends on the cross-sectional variation in the baseline grade’s test scores, as well as the comparability of test score gains across different parts of the learning distribution within each country. This comparison is also fragile because, if test scores are normally distributed, a 1 SD gain throughout the distribution should always imply that children at the 75th percentile in Grade 3 “know” almost the same as children at the 25th percentile in Grade 6. Although this is indeed the case for Pakistan and Florida, it is generally not true in the Young Lives countries. In India, Vietnam and Peru, children at the 25th percentile at age 12 know more than children at the 75th percentile at age 8, while the opposite is true in Ethiopia. The non-normality of these data could reflect that fact that children tested at the same age are in very different grades, further complicating cross-country comparisons.

### 3.2 Are test score gains due to “learning by aging?”

**Fig. 1:**
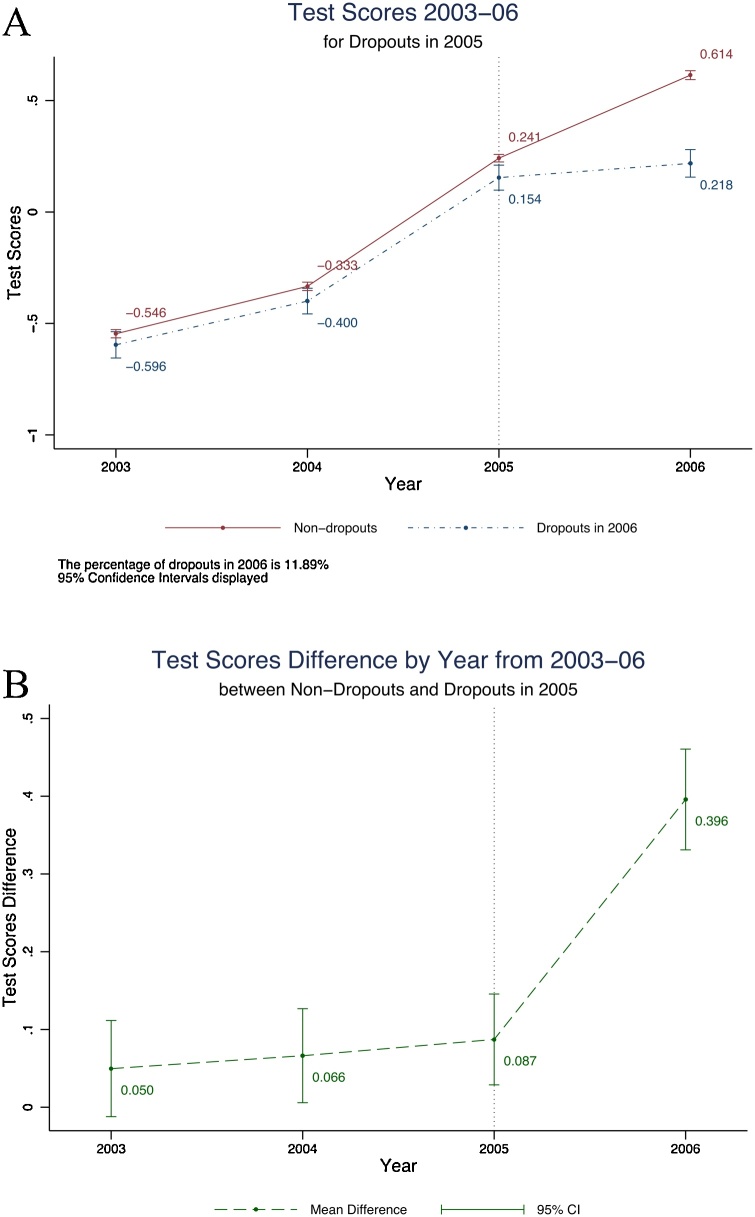
Learning trajectories for 2005 dropouts, non-dropouts and difference – combined test scores. Notes: Panel A shows test scores in every round for two groups of students in the full unbalanced school panel. The red solid line shows students who were enrolled in every year, while the dotted blue line shows test scores in every round for students who eventually dropped-out in the transition from primary to middle school. The last score for the dropout group reflects their scores when they were tested at home and have been out of school for one year. 95 % confidence intervals displayed for each year-group combination. The percentage of dropouts in 2006 is 11.89 %. Panel B shows the difference in test scores between both groups for each year and its corresponding 95 % confidence interval. Test scores refers to the mean across Urdu, English and Mathematics.

A second important question is the extent to which this learning reflects natural progression in vocabulary and Math skills due to “learning by aging” as opposed to “learning by schooling.” Fig. 1 Panel A plots test scores in every round for two groups of students in the (unbalanced) school survey. The red line shows students who were observed in every year. It is worth highlighting that the test score gains experienced by these students between 2003 and 2006 (of 1.16 SD) are almost identical to what we observe for the full school sample (1.15 SD). The blue line shows test scores in every round for students who eventually dropped-out in the transition from primary to middle school. The last score for this group therefore reflects their scores when they were tested at home and had been out of school for one year. Fig. 1 Panel B plots the yearly test score differences between these two groups with their respective 95 % confidence intervals.

**Fig. A2:**
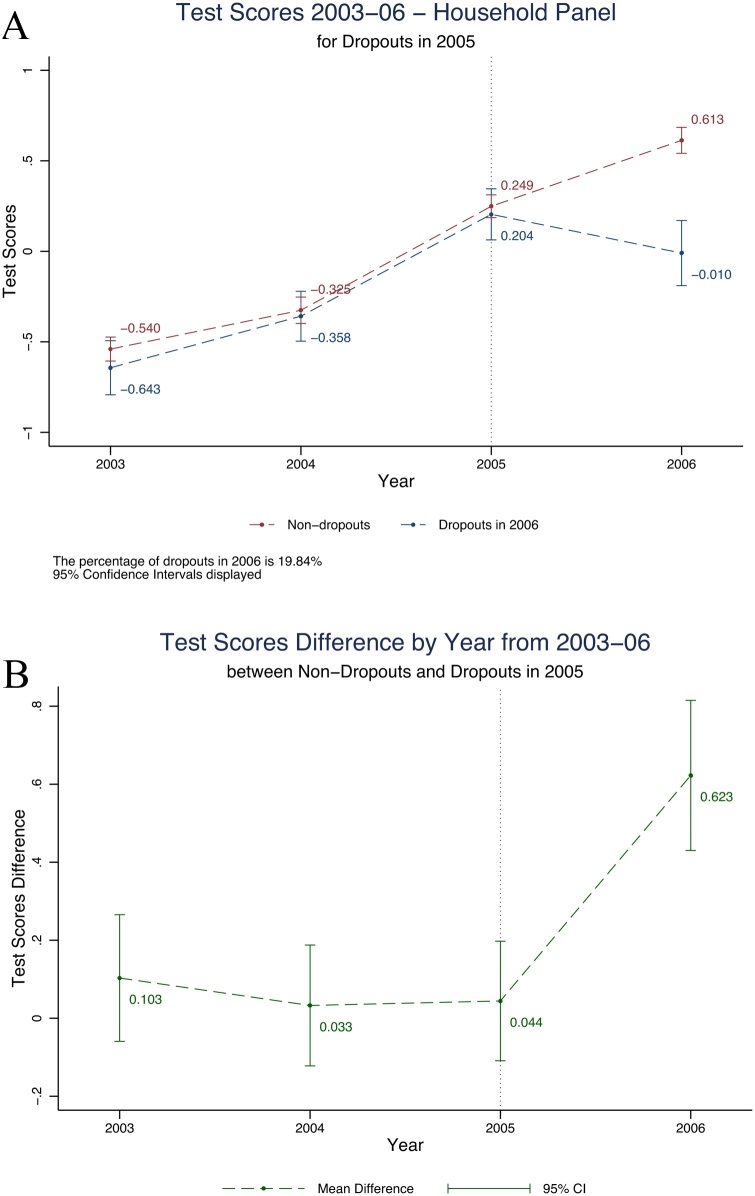
Learning trajectories for 2006 dropouts and non-dropouts – combined test scores (Household sample). Notes: Panel A shows test scores in every round for two groups of students in the household panel. The red line shows students who were enrolled in every year while the blue line shows test scores in every round for students who eventually dropped-out in the transition from primary to middle school. The last score for the dropout group reflects their scores when they were tested at home and have been out of school for one year. 95 % confidence intervals displayed for each year-group combination. The percentage of dropouts in 2006 is 19.84 %. Panel B shows the difference in test scores between both groups for each year and its corresponding 95 % confidence interval. Test scores refers to the mean across Urdu, English and Mathematics.

Test score gains track each other very closely between 2003 and 2005. There is a small but imprecisely estimated difference of 0.05SD to 0.09SD in test scores levels in favor of the children who continued, but there are no differences in gains. However, immediately after children dropout, the test score differences increase to 0.40 SD from 0.09 SD in the preceding year. The analog of this figure for the household panel (Appendix Fig. A2) shows similar patterns prior to dropout, but starker differences after dropping out, as children who are no longer in school report lower test scores in the year after dropping out. This striking finding suggests that children who dropout were learning no less than those who chose to continue. It is also an indication that school continuation remains an important source of inequality. The children who dropped out were more likely to come from less wealthy households with lower parental education (on a standardized asset index, the children who dropped out were 0.32 SD below those who continued, 12 % of their mothers and 40 % of their fathers had completed at least primary education compared to 24 % of mothers and 55 % of fathers for those who continued).

Table 4 shows the regression equivalent to Fig. 1 using the (unbalanced) school sample. We present four specifications, which differ in how we treat persistence in learning and how we treat dropouts. First, in columns 1 and 2, we present the association between dropping out and level test scores, either by examining the impact of dropping out in Round 4 (Column 1) or by allowing dropouts to have different test score gains in each year (Column 2), even before they dropout. Specifically, Column 1 estimates:

$$y_{it}=\beta_{0}+\beta_{1}\left(year\;2004\right)+\beta_{2}\left(year\;\;2005\right)+\beta_{3}\left(year\;\;2006\right)+\beta_{4}(Dropout\;05-06)+\varepsilon_{it}$$

while Column 2 estimates:

$$y_{it}=\beta_{0}+\beta_{1}\left(year\;\;2004\right)+\beta_{2}\left(year\;\;2005\right)+\beta_{3}\left(year\;\;2006\right)+\beta_{4}\left(Dropout\;Group\right)\times \left(year\;\;2004\right)+\;\beta_{5}\left(Dropout\;Group\right)\times \left(year\;\;2005\right){+\beta }_{6}\left(Dropout\;Group\right)\times \left(year\;\;2006\right)+\varepsilon_{it}.$$

Here, “Dropout 05−06” is an indicator variable equal to 1 if child dropped out between years 2005 and 2006 and *t* is 2006, and equal to 0 otherwise, including for all other years. “Dropout Group” is a time-invariant indicator variable equal to 1 for children who dropped-out between 2005 and 2006. These regressions are clustered at the child-level, since there are multiple observations per child. Columns 3 and 4 re-run these regressions, but we now allow the test score levels in time *t* to depend on test scores in *t-1*, using the frequently-used value-added specification. Specifically, the two specifications above include an additional term, $\beta_{1}y_{it-1}$, which accounts for the persistence of past test scores year-to-year so that the β coefficients can be interpreted in terms of yearly test score changes. While this specification better captures test score dynamics in our sample, it also reduces the data that are available by dropping 2003 test-scores where lags are not available, as well as any other individual with gaps in the panel.

**Table 4:** Test scores gains over the years, (imperfect) learning persistence, and dropouts.

	Dep. Var: Mean Test Scores			
	(1)	(2)	(3)	(4)
2004 Indicator	0.21***	0.22***		
	(0.035)	(0.036)		
2005 Indicator	0.79***	0.79***	0.35***	0.35***
	(0.028)	(0.028)	(0.0096)	(0.010)
2006 Indicator	1.18***	1.17***	0.36***	0.35***
	(0.042)	(0.041)	(0.011)	(0.011)
Dropout Indicator 2005−06	−0.45***		−0.35***	
	(0.055)		(0.031)	
Dropout Group		−0.095*		−0.057*
		(0.044)		(0.027)
2004 # Dropout Group		−0.016		
		(0.039)		
2005 # Dropout Group		−0.040		0.0055
		(0.044)		(0.036)
2006 # Dropout Group		−0.36***		−0.29***
		(0.057)		(0.042)
Test Score at (t-1)			0.71***	0.71***
			(0.0059)	(0.0060)
Constant	−0.78***	−0.78***	0.018	0.022*
	(0.056)	(0.056)	(0.0098)	(0.0099)
District Fixed-Effects	Yes	Yes	Yes	Yes
Observations	47,099	47,099	28,898	28,898
Adjusted *R*^2^	0.208	0.208	0.612	0.613

Notes: This table uses the full unbalanced school sample and is the regression analog of Fig. 1 (although controlling for district fixed effects, so estimates slightly differ). It shows the regression results of test scores in year *t* on year indicators and dropout indicators. Test scores refers to the mean across Urdu, English and Mathematics. The four specifications estimated differ in how persistence in learning and dropouts are treated. “Dropout Indicator 05−06” is an indicator variable equal to 1 if child dropped out between years 2005 and 2006 and *t* is 2006, and equal to 0 otherwise, including for all other years. “Dropout Group” is a time-invariant indicator variable equal to 1 for children who dropped-out between 2005 and 2006. Columns 1 and 2, present the association between dropping out and level test scores. Columns 3 and 4 re-run these regressions but allow the test score levels in time *t* to depend on test scores in *t-1* using the value-added specification. Standard errors clustered at the village level are in parentheses.

* *p* < 0.05, ** *p* < 0.01, *** *p* < 0.001.

Across all these specifications, the basic message remains the same. Tests score gains are significantly lower in the year that children dropout. These differences range from -0.29 SD (when we allow for the gain coefficient to vary by year and include the lagged test score in Column 4) to -0.45 SD, when we examine level test-score differences in Column 1. The coefficient is always statistically significant at the 99 % level of confidence. Children who dropout have lower test scores at baseline (although precision is lower given the small sample, the estimates range from -0.06 SD to -0.1 SD). There is no evidence, that conditional on lower test scores levels, their test score growth is different in any of the years prior to dropout. This provides evidence in favor of the parallel trends assumption required for the validity of this difference-in-difference approach. The finding is also novel in its own right—consistent with what we find, studies thus far have shown that test scores are correlated with dropping out—but have not examined the association between test score trajectories and dropping out. Finally, and we return to this later, there is clear evidence of imperfect persistence of learning from year to year. If learning perfectly persisted, the coefficient on lagged test scores would be equal to 1.

We conclude from these data that most of the gains we observe in test scores for children who are attending school are *because* they are in school and not because of natural gains as children age. There is evidence of gains on every tested item and some evidence that the relative gains across years are similar to what we see in other settings.^16^ Finally, children who stay in school learn more than those who dropout, and this difference in test-score gains emerges only in the year of the dropout. Of course, these data could reflect unobserved changes in family circumstances that are also correlated with test-score gains. However, the lack of a clear pre-trend for dropouts lends some credence to the hypothesis that these are indeed causal estimates.

### 3.3 Variation in learning and test score convergence

**Fig. 2:**
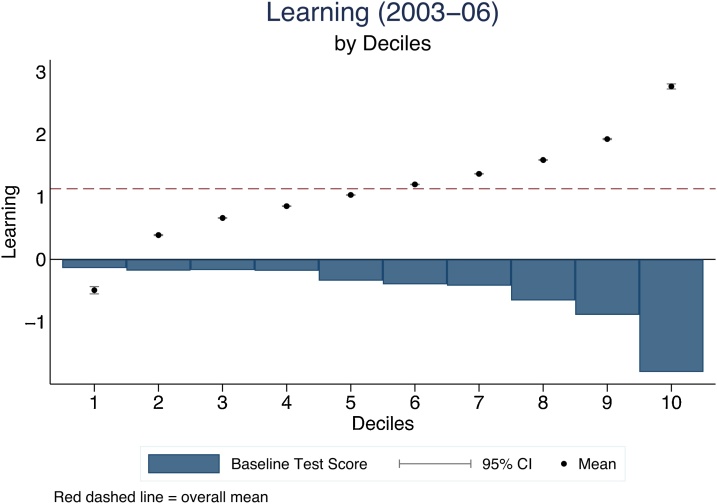
Four-year learning gains/losses by learning deciles. Notes: This figure plots test score gains from 2003 to 2006 by deciles of test score gains. Test scores refers to the mean across Urdu, English and Mathematics. Test score gains are defined as the difference in test scores between Grades 3 and 6. 95 % confidence intervals are also shown for each point but are very small. The bars show the test score in 2003 by decile with higher baseline test scores for those experiencing learning losses (i.e. decile 1). The red dashed line represents the overall test score gain mean. Test scores refers to the mean across Urdu, English and Mathematics.

The second part of our description of test score gains during primary school focuses on the variation in learning across the population. We first emphasize that there is substantial variation in how much children learn during their schooling years. Fig. 2 plots test score gains from 2003 to 2006 by deciles of test score gains between Grades 3 and 6. Standard errors are also plotted for each point (but are very small and hence not visible). The poorest 10 % of learners report *lower* test scores in Grade 6 compared to Grade 3. Beyond this lowest decile, all children gain over the primary school years, but the gains are highly variable. At the top end, children gained an impressive 2.8 SD over the duration of our data.

One concern that has become apparent in the recent literature on learning in LICs is that of “curricular mismatch.” This is the idea that children are taught according to a curriculum that is far too advanced for the average child (and maybe even the best performers), and therefore, children who are behind fall even further behind every year (See Beatty and Pritchett, 2015; Kaffenberger and Pritchett, 2020a; Duflo et al., 2011; and Banerjee et al., 2016 and 2017). Muralidharan et al. (2019) have demonstrated this pattern quite strikingly for children in middle school over the first few months of the schooling year. They have also shown that adaptive learning, where children are “taught where they are” rather than where they should be, can yield large learning gains in a short time period. These results are very similar to those discussed in an approach that has come to be known as “teaching at the right level,” pioneered by the Indian NGO, Pratham, and evaluated positively by Banerjee et al. (2017). Finally, Bau (2019) has demonstrated in the LEAPS data that private schools horizontally differentiate by setting different curricular levels.

**Fig. 3:**
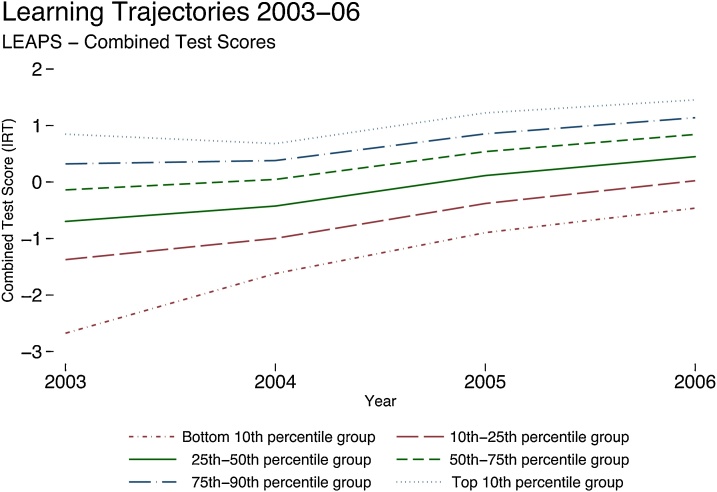
Convergence: Learning trajectories by percentile group from initial combined test scores. Notes: This figure shows learning trajectories by groups of baseline levels of test score performance during Grade 3–6 using the unbalanced full sample but restricting the graph for those who were observed in Grade 3 (2003). The graph shows averaged test scores across the three subjects tested (Appendix Fig. A3 shows the patterns for the 3 different subjects) for children at different test scores levels in 2003. That is, we have divided the children based on their baseline test scores in 2003 into six groups, as explained in the legend. Each line represents a group’s mean test scores over the rounds of testing.

**Fig. A3:**
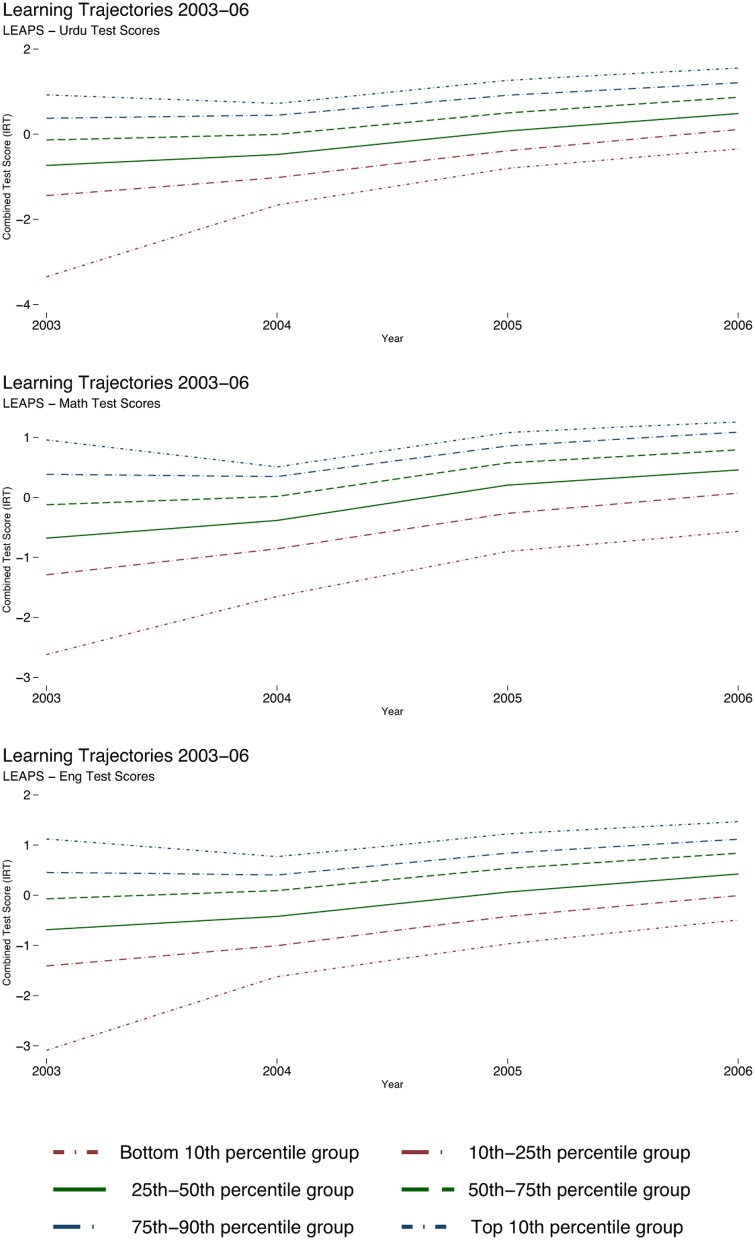
Convergence: Learning trajectories by percentile group from initial test scores by subject. Notes: This figure shows learning trajectories by groups of baseline levels of test score performance during Grades 3–6 using the unbalanced full sample but restricting the graph for those who were observed in Grade 3 (2003). The graph shows the patterns for the 3 different subjects (i.e. Math, Urdu, and English) for children at different test scores levels in 2003. That is, we have divided the children based on their baseline test scores in 2003 into six groups as explained in the legend for each subject. Every line represents their mean test scores over the rounds of testing.

While it is therefore clear that tailoring teaching to a child’s specific learning level yields positive dividends, there is no data thus far that allows us to look at learning trajectories by baseline levels during the primary schooling years in LMICs to see if the children who are behind indeed fall farther behind every year.^17^ Fig. 2 already suggests that children who gained the most reported the *lowest* test scores in Grade 3. Fig. 3 examines this pattern directly. Here, we have plotted test scores, averaged across the three subjects tested (Appendix Fig. A3 show the patterns for the 3 different subjects) for children at different learning levels in 2003. That is, we have divided the children based on their test-scores in 2003 into six groups, with the bottom representing the worst performing 10 %, the next group is the 10th to 25th percentile, followed by the 25th to 50th, 50th to 75th, 75th to 90th percentiles, and finally, the top 10 %. Every line represents their mean test scores over the rounds of testing.

There is no divergence in test scores in this figure. In fact, there is convergence. The difference between the bottom and top 10 % is 3.52 SD in 2003, which narrows sharply to 1.92 SD in 2006. This difference is not just across the bottom and top 10th percentiles. It reflects a gradual reduction of the baseline differences across all percentile groups. We can also confirm that the overall variance of test scores (which is 1 SD in 2003) has declined by 2006 to 0.98 SD. It is not only the case that children who are performing worse in 2003 are gaining more, but also overall inequality in learning is (weakly) decreasing between Grades 3 and 6. This is similar to recent findings from the United States—most test score divergence by race happens before primary school with stable gaps during the primary schooling years (Carneiro and Heckman, 2002).

#### 3.3.1 Robustness to measurement error

One potential complication for interpreting Fig. 3 in is the fact that measurement error in test scores will automatically lead to mean reversion and therefore conditional convergence. Suppose that every child actually has the same knowledge level in 2003 but that each child’s test score is measured with error. Then, the bottom quintile are children whose measurement error “shock” was highly negative, and the top quintile is those whose measurement error “shock” was highly positive. If there is no autocorrelation in measurement error and true ability gains are identical, the observed learning gain for the low performers in 2003 will be mechanically higher. Similarly, we should also expect the observed top quintile to have lower observed gains (if underlying gains are the same throughout the ability distribution) from year t to t + 1. However, the fact that the variance of test scores decreases over time provides initial evidence that this is not the entire story.

Table 5 presents an intuitive way to show that measurement error alone does not explain why children who are initially low performers report higher test score gains in our data. Here, we have formed quintiles by test scores in 2003, but then examined gains *only* between 2004 and 2006. If measurement error is idiosyncratic across years, it should affect the observed gains of a quintile calculated in 2003 from 2003 to 2004, but not the gains from 2004 to 2005 or 2004−2006. Again, we find greater learning between 2004 and 2006 for children classified in the bottom quintile in 2003. These gains are significantly lower than what we see when using all test scores, but these findings remain at odds with the idea that ex-ante poor performers learn less during the primary schooling years.

**Table 5:** Test scores over time and learning by quintile.

Quintiles by Test Score 2003	Stat	Test Score 2003	Test Score 2004	Test Score 2005	Test Score 2006	Learning (2006−04)	Learning (2006−03)
Quintile 1	*Mean*	−2.01	−1.34	−0.64	−0.26	1.10	1.75
	*N*	1471	1314	1249	1471	1314	1471
Quintile 2	*Mean*	−0.86	−0.55	0.00	0.35	0.92	1.22
	*N*	1471	1347	1275	1471	1347	1471
Quintile 3	*Mean*	−0.38	−0.14	0.37	0.67	0.83	1.05
	*N*	1471	1353	1312	1471	1353	1471
Quintile 4	*Mean*	0.04	0.19	0.67	0.95	0.77	0.91
	*N*	1471	1383	1332	1471	1383	1471
Quintile 5	*Mean*	0.62	0.58	1.09	1.33	0.77	0.71
	*N*	1471	1358	1336	1471	1358	1471
All	*Mean*	−0.52	−0.24	0.31	0.61	0.88	1.13
	*N*	7355	6755	6504	7355	6755	7355

Notes: This table uses the full unbalanced sample but is restricted to children observed in 2003, since new children in years 2004−06 cannot be classified in quintiles by 2003 test scores. Test scores refers to the mean across Urdu, English and Mathematics. Quintiles by test scores in 2003 are estimated only for those observed in 2006 as their test score in 2006 is needed to estimate their learning. For each quintile, the gains between 2004−06 and 2003−06 are shown. The table shows that measurement error alone does not explain why children who are initially low performers report higher test score gains in our data.

This procedure addresses mean reversion due to measurement error, but the gains are generally not equal to the true learning gains by baseline scores due to misclassification in the quintiles.^18^ This misclassification results in bias in the estimates of gains by quintile, but the direction of the bias is unclear since it will depend on how true ability gains change across the Grade 3 test score distribution. A more structured way to address the misclassification and measurement error problems together is to use the quintile in year 1 as an instrument for the quintile in year 2 and then regress the change in scores between years 2 and 4 on this quintile. That is, for the second stage regression in a two-stage least squares strategy, we estimate:

$$\text{Δ}{\text{y}}_{\text{i},4-2}=\beta_{0}+\sum_{j}{\beta_{j}I}_{i}^{Q_{j}}+\varepsilon_{it},$$

where $I_{i}^{Q_{j}}$ is an indicator variable for belonging to quintile $Q_{j}$ in year 2 (2004), which we instrument for with an individual’s quintile in year 1 (2003). If we omit the constant, we get the average gain by quintile (instrumented). If we include the constant, we get the relative gain by quintile. We can also estimate a second stage regression with a single test statistic that captures convergence/divergence:

$$\text{Δ}{\text{y}}_{\text{i},4-2}=\beta_{0}+\beta_{1}y_{i,2}+\varepsilon_{it}.$$

In this second case, we instrument for $y_{i,2}$ with $y_{i,1}$. If $\beta_{1}<0$, this implies test scores are converging over time. If $\beta_{1}>0$, this implies that test scores are diverging over time.

The instrumental variables estimates are presented in Table 6, where Column 1 omits the constant to obtain the average gain by quintile, and Column 2 shows gains for each quintile relative to the omitted category, Quintile 1. Column 3 estimates the continuous version of this equation, recovering $\beta_{1}$ as the convergence/divergence parameter. Across these specifications, we again find convergence—children with higher test scores in 2003 learned less between 2004 and 2006.

**Table 6:** Learning convergence: IV correction for miss-assignment and measurement error.

	Dep. Var.: Mean Test Score Gains 2004−06		
	(1)	(2)	(3)
Test Score Quintiles 2004 = 1	1.32***		
	(0.067)		
Test Score Quintiles 2004 = 2	0.92***	−0.39	
	(0.23)	(0.29)	
Test Score Quintiles 2004 = 3	0.87***	−0.45**	
	(0.16)	(0.14)	
Test Score Quintiles 2004 = 4	0.73***	−0.59***	
	(0.10)	(0.15)	
Test Score Quintiles 2004 = 5	0.81***	−0.51***	
	(0.062)	(0.12)	
Test Score in 2004			−0.18***
			(0.021)
Constant		1.32***	0.87***
		(0.067)	(0.024)
Mauza Fixed-Effects	Yes	Yes	Yes
Observations	6755	6755	6755
Adjusted *R*^2^	0.163	0.163	0.191

Notes: This table shows the regression results of 3-year test score gains (2004−06) on test scores or quintiles by test score in year 2 (2004) but instrumenting them with test scores or quintiles by test scores in year 1 (2003). Test scores refers to the mean across Urdu, English and Mathematics. Quintiles are estimated only for those observed in 2006 who had test scores in 2003 and 2004 respectively. Column 1 omits the constant to obtain the average gain by quintile, and Column 2 shows gains for each quintile relative to the omitted category, Quintile 1. Column 3 estimates the continuous version of this equation. The negative coefficient of Test Scores in 2004 from Column 3 implies test scores are converging over time. Convergence is evidenced across specifications with children with higher test scores in 2003 learning less between 2004 and 2006. Standard errors clustered at the village-level appear in parentheses. * *p* < 0.05, ** *p* < 0.01, *** *p* < 0.001.

### 3.4 Role of household characteristics

We next explore other determinants of learning besides a child’s location in the test score distribution. Table 7 uses the unbalanced panel to regress test scores in year *t* on lagged test scores at *t-1*, along with parental education, average wealth over the rounds of the survey (measured through an asset index), age, sex, and whether the child dropped-out in 2005−06.^19^ We also include, in different specifications, a full set of village or school fixed effects to capture potential differences by geography and schools. Strikingly, household and child characteristics explain very little of the variation in test score gains

**Table 7:** Test scores on lagged test scores, child and household characteristics and fixed effects.

	(1)	(2)	(3)	(4)
	Test score _t_	Test score _t_	Test score _t_	Test score _t_
Test Score _t-1_	0.74***	0.73***	0.71***	0.66***
	(0.0058)	(0.0060)	(0.0064)	(0.0076)
Father Educ: <Primary		−0.0043	−0.0030	−0.0058
		(0.013)	(0.012)	(0.012)
Father Educ: >Primary to Higher Secondary		0.070***	0.074***	0.064***
		(0.0097)	(0.0097)	(0.0094)
Father Educ: Higher Secondary or Higher		0.13***	0.13***	0.11***
		(0.016)	(0.016)	(0.016)
Mother Educ: <Primary		0.0053	0.0047	−0.0044
		(0.011)	(0.011)	(0.010)
Mother Educ: >Primary to Higher Secondary		0.044***	0.042***	0.018
		(0.0098)	(0.0100)	(0.0098)
Mother Educ: Higher Secondary or Higher		0.085**	0.080**	0.020
		(0.027)	(0.027)	(0.028)
Average PCA Asset Index across Years		0.015***	0.016***	0.0046
		(0.0026)	(0.0026)	(0.0025)
Age in 2003		−0.0093***	−0.0099***	−0.012***
		(0.0027)	(0.0027)	(0.0028)
Dropout Group Indicator		−0.13***	−0.13***	−0.075***
		(0.015)	(0.015)	(0.017)
Female Indicator		0.034***	0.040***	0.050***
		(0.0075)	(0.0075)	(0.012)
Constant	0.26***	0.29***	0.031	−0.63*
	(0.0070)	(0.029)	(0.080)	(0.27)
Mauza Fixed-Effects	No	No	Yes	No
School Fixed-Effects	No	No	No	Yes
District Fixed-Effects	Yes	Yes	No	No
Observations	23,992	23,992	23,992	23,990
Adjusted R-squared	0.59	0.59	0.60	0.64
Within Adjusted R-squared	0.56	0.57	0.54	0.43

Notes: This table uses the full unbalanced panel to regress test scores in year *t* on lagged test scores at *t-1* along with parental education groups, average wealth across rounds, baseline age, sex and whether the child dropped-out in 2005−06. Test scores refers to the mean across Urdu, English and Mathematics. Specifications across columns include a full set of village or school fixed effects to capture potential differences by geography and schools. The Within Adjusted R-square measures the explanatory power net of mauza, school and district fixed-effects respectively. Household and child characteristics explain very little of the variation in test score gains after accounting for fixed effects. Standard errors clustered at the village-level are in parentheses. The base category for the father and mother education groups is no education. The average wealth measure ignores missing data in any given year. * *p* < 0.05, ** *p* < 0.01, *** *p* < 0.001.

As is true in much of the value-added literature, most of the variation in test score levels is explained by lagged test scores, which alone account for 56 % of the variation. Conditional on lagged test scores, household characteristics all enter with the signs we would expect (children with educated mothers and fathers and wealthier families gain more), but they explain little of the additional variation. One particularly striking result is the difference between parental education and parental wealth. In our data, the most beneficial household characteristics are having a father and a mother with secondary education (only 5% of our students have at least one parent with these characteristics), and relative to having parents with no education, having both parents with greater than secondary education would predict 0.21 SD higher value-added. This is approximately 50 % of average annual gains in the sample. In contrast, conditional on the other controls, wealth is less predictive of learning, with a 1 SD increase in wealth only predicting a 0.02 SD increase in value-added. Including village or school fixed effects only accounts for an additional 6% of the variation in test scores that we observe over this time.

#### 3.4.1 Robustness to alternative scaling

The fact that parental education matters but wealth does not is puzzling given an emphasis on the role of credit constraints in education, particularly in LICs. Suppose a parent is not educated but wealthy. Why can’t they “buy” the inputs provided by an educated parent on a tutoring market (for instance)? Given this potential puzzle and its implications, we were concerned that the weak correlation between (longer) 4-year test-score gains and two important characteristics —gender and family wealth— are a facet of the specific item weights generated by the Item Response procedure. This is an issue that has been raised in the literature on test score gains in school when Blacks are compared to Whites in the United States, where Bond and Lang (2013) have pointed out that the results are sensitive to test score scaling choices, implicit in the test construction.^20^ To address this, Bond and Lang (2013) developed a methodology to bound learning gains differences for groups over time by finding test score scale monotonic transformations that minimize and maximize differences in gains or even convert them into loses.

Yi Chang (2019) has developed the STATA module *scale_transformation* to implement this routine more generally, and the results from this bounding exercise are shown in Table 8. Table 8 shows the “worst” case (most extreme) bounds for the gender and household wealth learning gains between 2003 and 2006 in Columns 2 and 3 and compares them to the “raw” gap in our data displayed in Column 1.^21^ For gender, the bounds are positive but quite small, suggesting that test score gains (slightly) favor females by 0.01 to 0.03 SD. For household wealth, the bounds support a much wider range of meaningful differences that include no gap at all. This is a well-known problem in the Bond-Lang methodology, and therefore, in Columns 4 and 5, we have also presented the resulting gap from transformations that maximize the correlation and R^2^ of test scores over time, which helps to benchmark the wider bounds range against a likely transformation. In combination with the bounds, these estimates do not suggest that there are large gains among children with higher wealth. If anything, the evidence points towards small or no differences by the families’ wealth.

**Table 8:** Robustness to scaling transformations and likely transformation for wealth and gender gaps.

Groups	Gap Growth			Correlation	R-square
	Original	Min	Max	Max	Max
*Wealth Top vs Bottom Quartile*	−.0792	−.1185	.0829	−.1184	−.0094
*Gender (Female)*	.0097	.0047	.0277	.0049	.0176

Notes: This table compares the original 4-year learning gap for wealth and gender to those obtained from extreme monotonic transformations that maximize and minimize these gaps. The gap is defined as the coefficient on the variable for wealth or gender in the regression for year 1 minus the same coefficient in year 4. Specifications are similar to those in Table 7 and control for district fixed effects, age in 2003, parental education, and a dropout group indicator. Additionally, the wealth gap controls for gender, and the gender gap controls for wealth. The table also provides likely transformations, those that maximize correlation and R-square of test scores in year 1 and 4, to help benchmark the results. Wealth quartiles are constructed from the PCA of mean household assets across years. Max and Min Gap Growth discard very unlikely transformations, specifically those with skewness outside [-2,2] and/or kurtosis outside [0,10]. For computational speed and efficiency reasons, convergence is assumed after 15 iterations, and monotonicity is only checked up to a finite number of possibilities (46,735 different IRT scores that came from 4 rounds of surveys). Furthermore, the program allows the gap to reverse. This efficiency gain and flexibility might yield, in rare instances, results in the opposite direction of the intended max/min optimization. These unlikely results are discarded for this exercise.

## 4 Fragile learning

Although we find that there are test school gains over the course of primary schooling, as well as test score convergence, our results do not necessarily imply that the school system in Punjab is well-functioning. In all three tested subjects, there are basic tasks that children cannot perform correctly by the time they are in Grade 6. In English, 54 % cannot write the word "girl"; 80 % cannot construct a sentence with the word "play." In Mathematics, 49 % cannot subtract 238−129, and 74 % cannot multiply 417 and 27. Children find it hard to form plurals from singular forms in Urdu, and 55 % cannot form a grammatically correct sentence with the word "*karigar*" (which means “workman”).^22^ For the 22 % of children in our household sample who will not continue their schooling past Grade 6, these are the skills they will have to bring to their work environment.^23^ The challenge is how to rationalize this poor level of performance across subjects by Grade 6 with the facts that (a) the fraction of children answering questions correctly increases with every grade (attributable to being in school, rather than ‘learning by aging’), and (b) test score gains are consistently higher among those with the lowest scores in Grade 3. That, in turn, raises difficult questions about test score measurement and what the literature has euphemistically termed "mean reversion."

### 4.1 Gains versus value-added specifications

We start by discussing how gains versus value-added specifications can yield seemingly conflicting patterns, and how this is related to low persistence in learning. Suppose we estimate a "gain" specification of the form, $y_{it}-y_{it-1}=\;\beta_{0}\;+\;\beta_{1}X_{it}+\varepsilon_{it}$, where $X_{it}$ could be individual or household characteristics. Then, the estimated $\beta_{1}$ are usually small—test score gains are weakly correlated with household characteristics.^24^ We can also estimate a "value-added" specification, $y_{it}\;=\;\gamma_{0}\;+\gamma_{1}X_{it}+\lambda y_{it-1}\;+\;\eta_{it}$, where the control for lagged test scores allows for imperfect persistence (λ<1). Typical estimates of λ in the value-added specification are between 0.5 and 0.7 rather than the 1 assumed in the gains specification. Consequently, when the $Cov(y_{it-1},X_{it})>0$, estimates of $\gamma_{1}$ are considerably larger than estimates of $\beta_{1}$.

**Fig. A4:**
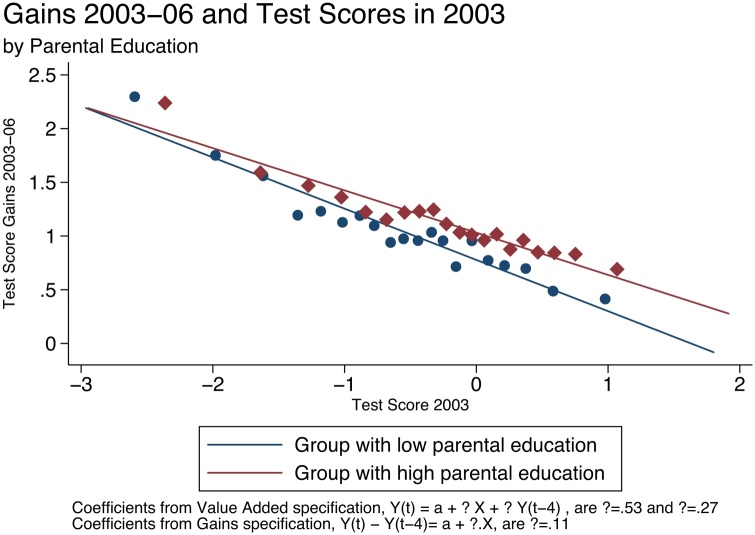
Linear fit of 4-years gains and baseline test scores. Notes: High and low parental education groups are constructed from the maximum level of education across father and mother for each child. High education groups are those where the most educated parent has completed more than primary school, and low education groups are families where the most educated parent reports 0 years of schooling. In our data, 22 % of parents fall in the first category, and 29 % fall in the second category. The figure shows, for each group, 20 quantiles in each group and the corresponding linear fit using Cattaneo et al. (2019) binscatter command in Stata. For clarity, we have excluded 2% of the observations with test scores higher than +2 SD or lower than -3 SD. We have also reported coefficients from the value-added and the gains specification where all data are included. The value-added specification shows that children who started off at the same score in 2003 gained 0.27 SD extra if they were in “high” education households. The gains specification shows that, on average, children from high education households gained an additional 0.11 SD between Grades 3 and 6.

As a concrete example, low persistence implies that children with more educated parents will gain less in a specification that assumes λ=1 because they have a higher test score to begin with.^25^ For instance, Appendix Fig. A4 plots test score gains ($y_{i,2006}-y_{i,2003})$ over the 4 years of our data against baseline scores in 2003 for groups with low and high parental education. Gains in both groups are negatively correlated with baseline scores because λ <1. For parental education, the gains specification estimates $\beta_{1}=0.11,$ while the value-added specification estimates $\gamma_{1}=0.27$ for the same parental education indicator. This difference arises because children with more educated parents had higher test scores in 2003.

If test scores are a surrogate welfare measure, arguably the gains specification ($y_{it}-y_{it-1})$ is more attractive. If adult welfare increases with test scores in Grade 6, the fact that the gains between Grades 4 and 6 are equal across high and low parental education groups is surely what matters. Alternatively, if we are interested in the production function of education, the value-added specification may be more appropriate, as $y_{it-1}$ stands-in for omitted child ability and cumulative investments as of *t-1*. Indeed, Andrabi et al. (2011) showed that the value-added specification precisely replicated the gains among children switching to a private school, even though the gains specification yields an approximately 0 coefficient on private schooling. This result foreshadowed a large and growing literature using value-added models to estimate the productivity of teachers (Chetty et al., 2014; Bau and Das, 2020) and schools (Angrist et al., 2017; Andrabi et al., 2020).^26^ Using $y_{it-1}$ as a control to address omitted variable bias is therefore well-established in the production function literature and in RCTs, where it serves to increase precision, given that $Cov(y_{it-1}\;,\;X_{it}$) = 0 (Bruhn and McKenzie, 2009).

Yet, when learning trajectories are themselves the *focus* of research, the difference between the gains and the value-added specifications can create confusion, and therefore the interpretation of “λ” itself becomes a valid object for further enquiry. Indeed, as we discuss in more detail in Appendix B, using a value-added style specification (similar to Muralidharan et al., 2019) would lead us to find test score divergence (larger test score growth for those with initially high test scores) rather than convergence under the (strong) parametric assumption that persistence is identical across the test score distribution.

### 4.2 Fragile learners

We believe that studying test score trajectories requires us to have a pedagogical interpretation for the mean reversion parameter,  λ, particularly if we want to rationalize low levels of accumulated knowledge as arising from low rates of learning. We present a heuristic argument that low levels of levels of test scores cannot be equated to low rates of learning – they may reflect rapid learning followed by reversals. Therefore, the reasonable assumption that *the likelihood of answering an item correctly is always (weakly) increasing with time for all students* is incorrect. We present this argument in three parts. First, we show that a sizeable fraction of our sample experiences year-to-year learning losses. Second, we introduce the idea of “fragile learners” and show that this is not just due to guessing in multiple choice questions. Third, we show that the gains versus value-added specification choice has fundamental implications for modelling convergence in knowledge in these data. We emphasize that the concept of fragility is also built into the item characteristic curve –the idea that there are portions of the curve where ability is such that there is a *probability* of answering a question correctly implies some stochasticity in the learning process (or at least how students translate knowledge into answering questions). The question is how to think concretely about this stochasticity and its implications.^27^

#### 4.2.1 Year-to-year losses

In the value-added specification, test score levels increase because low persistence is balanced by additional inputs into the production function. Test score losses across years must then reflect a combination of very low levels of inputs and/or low persistence. Such losses are surprisingly frequent; in our data, 7% of children reported lower test scores in Grade 6 compared to Grade 3. More tellingly, the fraction of child-years where we see an absolute loss in test scores across consecutive years is considerably higher at 20 %. Every year, a fifth of children are measured as “knowing” less than they did the year before.

#### 4.2.2 Fragile learners, guessing, and measurement error

These losses cannot just be attributed to guessing in multiple choice questions or the concavity of learning trajectories, where additions to knowledge require greater inputs at higher levels. As a specific example, consider two questions in Mathematics. Children are given two boxes: one with 4 crescent moons and one with 8, and subsequently asked to circle the one with more objects. For the second one, children are given a box with 2 stars and asked to circle the number that matches the number of stars in the box. This is a difficult question for our sample, and by Grade 6, 27 % and 22 % get it wrong. Because there are 2 and 4 options respectively, guessing would imply that the fraction who "know" how to do this is even lower. A test with *only* these two questions administered in Grade 6 could lead us to conclude that the accumulation of counting skills is very slow during the primary years.

But this inference is complicated by two additional pieces of data. First, of the 25 % of children who cannot count stars in Grade 6, 82 % can add 3 + 4; 72 % can add 9 + 9+9, and 55 % can multiply 4 × 5. Children who can perform more complex tasks that involve counting still may not know how to count as required by the first two questions. More surprisingly, among those who could not count the stars in Grade 6, between 40 % and 50 % correctly answered these questions in Grade 5, and between 37 % and 46 % correctly answered the question in Grade 3. These are considerably higher than the fraction we would expect from pure guessing, suggesting that they *knew how to answer these questions but then subsequently "forgot."*

This example leads us to introduce the idea of “fragile learners,” who we define as children whose learning on a specific question does not follow a (weakly) monotonic trajectory. Appendix Table A5 examines year-by-year performance on specific questions, where each row is a question-specific learning trajectory. A child whose row reads (0,0,1,1) answered the question correctly in years 3 and 4, but not in years 1 and 2; a child whose row reads (1,0,0,1) answered the question correctly in years 1 and 4 but not in between. We divide children into four categories: (1) "always" and (2) "never learners," who could answer the question correctly in every year or never managed to answer correctly; (3) "robust learners," those whose trajectories show (weakly) monotonic progression starting from a point where they could not answer the question and (4) "fragile" learners, or those whose trajectories show regression at some point.

**Fig. 4:**
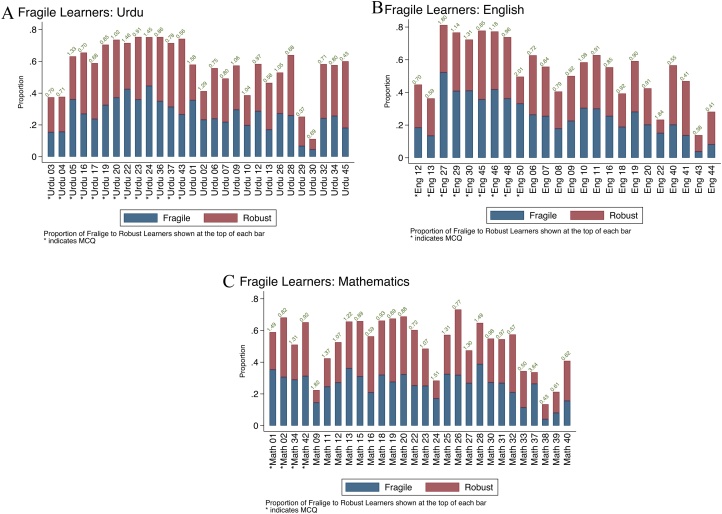
Proportion of fragile and robust learners by subject. Notes: This figure examines the proportion of students from the balanced panel (i.e. those observed every year, N = 6038) that, for each anchoring question asked every year, can be classified, based on the pattern of their correct/incorrect answer, as: (i) robust learners: those whose trajectories show (weakly) monotonic progression starting from a point where they could not answer the question; and (ii) fragile learners: those whose trajectories show regression at some point. The proportion of fragile to robust learners is shown at the top of each bar. An asterisk before the question indicates that the item was a multiple-choice questions (MCQ). The missing proportion corresponds to always or never learners, those who always or never answered correctly a given item.

Fig. 4 shows that robust learners range from 10 % to 37 % for the anchoring items that were asked in every year. This is in line with the average gain that we see. Interestingly, depending on the question, as a fraction of robust learners, fragile learners range from 40 % to 185 %. On average, as many children learn and forget how to answer a question as children who learn how to answer a question and are then able to answer it correctly in the subsequent year.

Fragility could be attributed to children guessing correctly in a multiple-choice question (MCQ) one year and incorrectly in the next, and indeed fragile learners are a higher fraction of robust learners for MCQs, a feature that is also captured in the higher guessing parameters for these items.^28^ Interestingly, this is not the only —or even the main— reason for fragility. Many questions do not follow an MCQ format (shaded in orange). Take (the non-MCQ) Math Item 9, which asks the child to add 3 + 4. The majority, 77 %, knew how to answer this question by Grade 3 and continued to know how to do so. Among the remaining children, 8% learned this in a way that once they had answered it, they continued to answer correctly. But 15 % "learned" it in a way that they could answer it correctly in some years, but not in others. These patterns do not ascribe to a model where children who do not know a concept or question learn it and then can answer it correctly forever. Instead, correct answers on specific items reflect a complex hilly landscape with peaks and valleys.

#### 4.2.3 Implications of fragility for convergence

Our inadequate understanding of fragility and mean reversion affects our understanding of test score trajectories. Fig. 2, which shows test score gains across 4 years, demonstrates losses for the lowest decile. As we have shown, the natural interpretation that children who were poor performers in Grade 3 also learned very little is incorrect; in fact, children who learned the least across 4 years were the best performers in Grade 3. This figure suggests that the problem is at the top — the best performers are not able to progress— rather than at the bottom. Again, the question of whether this is entirely due to mean reversion or curricular design is critical for researchers’ conclusions.

The fundamental problem is that we cannot tell from these data alone whether children at the top or the bottom are in fact learning less, since the answer depends on our assumptions about the constancy of the persistence parameter across the test score distribution. Assuming the persistence parameter is constant treats imperfect persistence as a "natural dynamic," independent of the pedagogic process. This assumption may entirely miss the point. Persistence may itself be a *function of the pedagogic* process and may vary across different students due to the pedagogic process. Unfortunately, with our data –as well as virtually all other data from low-income countries– lower levels of persistence cannot be observationally separated from lower levels of learning.

Our preliminary investigation suggests that learning trajectories are extremely complicated and unpacking this complexity is a critical task for education specialists moving forward. Thus far, our heuristic definition of fragile learning and its implications for test score trajectories lack a formal exploration, both in terms of the underlying statistics and the pedagogic content. We see this area as fertile grounds for further research, particularly if more long-term panels of test scores become available.

## 5 Discussion and conclusion

Our findings shed light on three patterns that are widely believed to characterize education in LICs. The first is that children learn very little and “flat” learning trajectories lead children from low-income countries to consistently test more than 1SD below those from high-income settings. The second argues that low learning is closely tied to pedagogical styles and suggests that because the grade-level curriculum is far more advanced than what children know, poor performers fall back relative to high achievers as they proceed through school. Finally, the third argues that education is for the elite, and therefore, children from wealthier backgrounds learn significantly more.

Our findings suggest that more nuance is warranted. It is certainly the case that children do not know a lot in Grade 6, particularly in Mathematics and English, but there is also clear evidence that they have learned between Grades 3 and 6, increasing performance by 20–30 percentage points on specific items. Our analysis of dropouts suggests that remaining in school adds considerable value, and therefore retention policies remain important to improve learning and equity in our setting.

Our data also suggest that schools are an equalizing force in these settings, in that children with initially low scores experience higher gains over the primary schooling years and the overall variance of the test score distribution does not increase. It is possible that the patterns in middle school are different, like in Muralidharan et al. (2019), although we have argued that the difference between their results and ours arises from conceptually different specifications. This convergence also does not detract from the fact that adaptive pedagogy that is targeted to the actual knowledge of a child can increase test scores; both children who were performing poorly and those at the higher ends of the spectrum may benefit from a more tailored approach. Bau (2019) shows how private schools differentiate through focusing on different types of students in our context.

Finally, parental wealth and child gender have little association with test score gains, although there are clear associations with parental education. However, the characteristics available in these data still only account for at most 6% of the variation in test scores, conditional on past test scores that we observe. This is consistent with emerging evidence from the U.S. that gaps in test scores have already developed by the time that children enter primary school (in our data, there are consistent differences in test scores when first measured by family background), and they do not expand much farther.

The unique data on learning trajectories available through the LEAPS project helps us rationalize this “positive” message with the low accumulation of skills in Grade 6 through the novel concept of “fragile learning.” We have shown that rather than slow but steady progression on specific questions, children may gain rapidly but then show no further increases or reversals. We have also shown that the fraction of children whose learning is fragile is as high as those who learn in a robust, monotonic fashion. It is this fragility, usually captured in a (low) persistence parameter that is central to our understanding of learning trajectories in low-income countries. We do not know whether such learning trajectories reflect differential effort in test-taking (Akyol et al., 2018), extreme sensitivity to testing and other environmental conditions (Mendell and Garvin, 2005), or a fundamental feature of the educational process.

This is one area where more research is needed with better longitudinal data using equated test scores over the primary schooling years. Panel datasets from schools in low-income countries that have tested children each year through the primary schooling years in a psychometrically valid fashion that allows for a comparison are exceedingly limited (one example with 3 years of data follows children from Grades 1–3 in South Africa^29^). Such data would allow researchers to examine critical questions about the link between the educational process, low persistence, and differential rates of learning across the test score distribution, helping to deepen our understanding of the concept of fragility that we have advanced here.

## Figures and Tables

**Table A1:** Comparing IRT test scores with model using restricted questions and fixed year 1 parameters.

		(1)		(2)		(3)	t-test	t-test
		IRT 4yrs		IRT 4yrs Restricted Qs		IRT 4yrs All Qs Fixed Yr 1 & Varying Params	Difference	Difference
Variable	N	Mean/SE	N	Mean/SE	N	Mean/SE	(1)-(2)	(1)-(3)
Combined Theta Year 1	12,109	−0.550	12,109	−0.535	12,109	−0.108	−0.015	−0.441***
		[0.009]		[0.009]		[0.010]		
Combined Theta Year 2	12,806	−0.339	12,806	−0.341	12,806	0.115	0.003	−0.453***
		[0.009]		[0.009]		[0.010]		
Combined Theta Year 3	12,123	0.235	12,123	0.231	12,123	0.735	0.004	−0.500***
		[0.008]		[0.008]		[0.009]		
Combined Theta Year 4	10,067	0.567	10,067	0.554	10,067	1.091	0.013	−0.524***
		[0.010]		[0.010]		[0.011]		
Combined Learning (2006−03)	7355	1.129	7355	1.102	7355	1.213	0.027*	−0.084***
		[0.010]		[0.011]		[0.011]		
English Theta Year 1	12,109	−0.528	12,109	−0.517	12,109	−0.102	−0.011	−0.426***
		[0.011]		[0.011]		[0.012]		
English Theta Year 2	12,806	−0.318	12,806	−0.323	12,806	0.118	0.005	−0.436***
		[0.010]		[0.010]		[0.010]		
English Theta Year 3	12,123	0.201	12,123	0.198	12,123	0.658	0.003	−0.457***
		[0.009]		[0.009]		[0.009]		
English Theta Year 4	10,067	0.542	10,067	0.534	10,067	1.016	0.008	−0.473***
		[0.011]		[0.011]		[0.011]		
Math Theta Year 1	12,109	−0.502	12,109	−0.478	12,109	−0.087	−0.024*	−0.414***
		[0.009]		[0.010]		[0.011]		
Math Theta Year 2	12,806	−0.335	12,806	−0.339	12,806	0.090	0.004	−0.426***
		[0.010]		[0.010]		[0.011]		
Math Theta Year 3	12,123	0.268	12,123	0.261	12,123	0.761	0.007	−0.493***
		[0.009]		[0.009]		[0.010]		
Math Theta Year 4	10,067	0.526	10,067	0.505	10,067	1.045	0.021	−0.519***
		[0.011]		[0.011]		[0.012]		
Urdu Theta Year 1	12,109	−0.619	12,109	−0.611	12,109	−0.135	−0.009	−0.484***
		[0.011]		[0.011]		[0.012]		
Urdu Theta Year 2	12,806	−0.362	12,806	−0.362	12,806	0.137	−0.001	−0.499***
		[0.011]		[0.011]		[0.012]		
Urdu Theta Year 3	12,123	0.237	12,123	0.234	12,123	0.788	0.003	−0.551***
		[0.009]		[0.009]		[0.010]		
Urdu Theta Year 4	10,067	0.632	10,067	0.623	10,067	1.213	0.009	−0.581***
		[0.010]		[0.010]		[0.011]		

Notes: This table shows the differences between IRT-estimated tests scores levels and gains used throughout the paper (i.e. column 1) versus: those recomputed after dropping the 19 questions where vertical equating seems to fail in column (2); and, in column (3), those recomputed using all questions but using year 1 parameters for all but varying parameters for those 19 items where vertical equating seems to fail. It shows no appreciable difference in test scores or gains between (1) and (2). Similarly, (3) shows significant yearly test score level differences with (1) but very similar yearly gains with only a slightly larger 4-year learning. The value displayed for t-tests are the differences in the means across the groups. Standard errors are robust. ***, **, and * indicate significance at the 1, 5, and 10 percent critical level.

**Table A2:** Learning simulation for random absence and misclassification.

Present in	Actual Presence in our Sample (%)	Presence w/ Random Absence 10 %	Presence w/ Random Absence 15 %	Presence w/ Random Absence 20 %	Presence w/ Random Absence 10 %			Presence w/ Random Absence 15 %			Presence w/ Random Absence 20 %		
					Misclass 3%	Misclass 5%	Misclass 7%	Misclass 3%	Misclass 5%	Misclass 7%	Misclass 3%	Misclass 5%	Misclass 7%
*Only 1 year*	22.4	2.7	5.8	9.7	6.7	9.4	12.1	10.1	13.0	15.9	14.3	17.4	20.4
*2 years*	27.4	24.3	32.6	38.7	29.3	32.0	34.2	36.2	38.1	39.6	41.1	42.3	43.1
*All 3 years*	50.2	73.0	61.6	51.6	64.0	58.6	53.7	53.7	48.9	44.5	44.5	40.3	36.5

Notes: Simulations use a total sample size of 16,428 across all years (the same as the number of unique students across all 4 rounds in the LEAPS sample). For simulating misclassification, this sample size grows every round. The average of 1000 simulations is shown above. Absence selection is random and independent each year. Misclassification selection in year t + 1 is random and conditional on being observed in year t and being observed at least once prior to year t (otherwise, it would not be misclassification but rather a “newly” observed student). Misclassified individuals are duplicated as new individuals, assigned a new unique ID, and are marked as not being observed every year prior to the misclassification period. Their original record for the year they were misclassified is then corrected to not observed. Misclassification rates are applied over the full sample size (not only those eligible based on being observed in year *t* and having been observed at least once before).

**Table A3:** Sample of children by number of years observed, child and household characteristics and mean learning (Household sample).

N Years Observed	N Child-Year Obs.	% Obs.	N Unique Children	Female Proportion	Age (2003)	Avg Days Absent (last 30 days)	% Fathers w Primary Edu. or Less	% Mothers w Primary Edu. or Less	HH Assets PCA	Avg Annual Learning
4	2556	71.62	639	0.45	9.6	1.8	48.3	77.25	0.04	0.38
3	741	20.76	247	0.45	9.7	2.0	46.9	74.23	−0.03	0.34
2	214	6.00	107	0.47	10.0	1.8	44.1	73.53	−0.17	0.28
1	58	1.63	58	0.34	9.8	1.7	59.4	90.63	−0.49	–

Notes: This table uses the full household sample. The number of years categories are exclusive, so children observed for 1 year are not counted again in other categories. Age in 2003 is estimated for those not present in that year. Average annual learning is defined as the mean of learning between every year present. If there are 2- or 3-year gaps, then learning is divided by the number of years. Father and mother education groups (used to construct % of fathers and mothers with primary education or less) and household assets are not available for every child as these data was only collected for a subsample of children that have test scores. The household assets PCA is the average of all years observed, ignoring missing data. The household assets PCA index is very highly correlated (corr = .96) with an index constructed using IRT on the same household assets (see Appendix Fig. A5 for details on how these two measures compare). Fathers, mothers, and household asset information used is from school survey to make it comparable with Table 2 and was cleaned to make it stable across years (see Appendix Table A8 for details on how these variables were cleaned).

**Table A4:** Learning for unbalanced, balanced, and household panels.

Variable	(1)		(2)		(3)		t-test	t-test	t-test
	Unbalanced Panel		Balanced Panel		Household Panel		Difference	Difference	Difference
	N	Mean/SE	N	Mean/SE	N	Mean/SE	(1)-(2)	(1)-(3)	(2)-(3)
*Learning 2003−06*	7355	1.129	6038	1.155	1406	1.101	−0.026*	0.028	0.054*
		[0.010]		[0.011]		[0.025]			
*Learning 2004−06*	8470	0.889	6038	0.884	1417	0.833	0.006	0.056**	0.051**
		[0.008]		[0.009]		[0.022]			
*Learning 2005−06*	8796	0.355	6038	0.344	1412	0.305	0.011	0.050**	0.040*
		[0.007]		[0.008]		[0.019]			
*Learning 2003−05*	8829	0.799	6038	0.811	1404	0.813	−0.012	−0.014	−0.003
		[0.008]		[0.009]		[0.019]			
*Learning 2004−05*	10,212	0.537	6038	0.539	1427	0.542	−0.002	−0.004	−0.003
		[0.006]		[0.007]		[0.015]			
*Learning 2003−05*	9890	0.258	6038	0.272	1450	0.284	−0.014	−0.026	−0.012
		[0.008]		[0.010]		[0.020]			

Notes: This tables compares learning for 3 different samples: (i) a balanced sample of 6038 children who were present in every year; (ii) the full unbalanced samples of children present in different years; and (iii) the household sample where the balanced proportion is higher. It shows that test score gains are very similar across all three samples, with statistically significant differences between the unbalanced/balanced panels when comparing against the household panel only when learning includes year 4. This is likely caused by the inclusion of testing dropouts at home and the fact that the balanced panel itself is a (slightly) selected group of children. The value displayed for t-tests are the differences in the means across the groups. Standard errors are robust and shown in brackets. ***, **, and * indicate significance at the 1, 5, and 10 percent critical level.

**Table A5:** Learning patterns by subject and items.

Notes: This table shows the proportion of students from the balanced panel (i.e. those observed every year; N = 6038) that, for each anchoring question asked every year, can be classified, based on the pattern of their correct/incorrect answer, as: (i) always learners: those who always answered correctly an item; (ii) never learners: those who never answered correctly an item; (iii) robust learners: those whose trajectories show (weakly) monotonic progression starting from a point where they could not answer the question; and (iv) fragile learners: those whose trajectories show regression at some point. The proportion of fragile to robust learners is shown at the bottom of the table. Non multiple-choice questions are highlighted in orange.

**Fig. A5:**
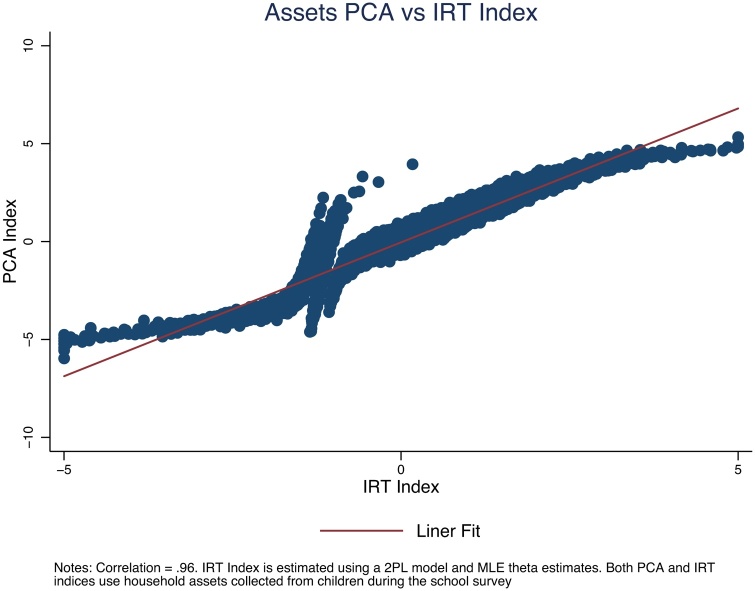
Scatter plot and linear fit of household assets PCA and IRT indices.

**Table A6:** Regression of test score in year t on yearly quintiles of performance and test score lags in year t-1.

	Dep. Var.: Test Score in Year *t*	
	(1)	(2)
Test Score (t-1)	0.55***	0.56***
	(0.031)	(0.032)
Quintile from Test Score Performance (t-1) = = 2	0.12***	0.12***
	(0.030)	(0.031)
Quintile from Test Score Performance (t-1) = = 3	0.23***	0.23***
	(0.046)	(0.047)
Quintile from Test Score Performance (t-1) = = 4	0.34***	0.34***
	(0.059)	(0.061)
Quintile from Test Score Performance (t-1) = = 5	0.43***	0.44***
	(0.067)	(0.069)
Year 2005 Indicator	0.38***	0.38***
	(0.034)	(0.035)
Year 2006 Indicator	0.45***	0.44***
	(0.038)	(0.039)
Dropout Group Indicator	−0.15***	−0.15***
	(0.020)	(0.019)
Constant	−0.54***	−0.28***
	(0.061)	(0.070)
Mauza Fixed-Effects	Yes	No
District Fixed-Effects	No	Yes
Observations	28,898	28,898
Adjusted *R*^2^	0.623	0.615

Notes: This tables replicates the results of Muralidharan, Singh and Ganimian (2019) value-added specification: $y_{it}=\;\beta_{0}\;+\lambda y_{it-1}+\sum_{q}\delta_{q}Q^{q}+\varepsilon_{it}$, where q sums over the quartiles of lagged test scores within a grade, and Q^q^ is an indicator variable equal to 1 if a student is in quartile *q*. To maintain comparability with other tables in this paper, we use quintiles rather than quartiles. This regression uses all-year IRT scores possible considering it includes lags. Quintiles are constructed within each year and might therefore vary across years for observations. As in Muralidharan, Singh and Ganimian (2019), identification is achieved because $y_{it}$ is computed across years, while quintiles are year specific. The omitted quintile indicator corresponds to the top quintile.

*p < 0.05, ** *p* < 0.01, *** *p* < 0.001.

**Table A7:** Yearly forward test score gains by test score and quintiles in previous year.

Groups	Stats	Quintiles					Total
		1	2	3	4	5	
*[-5 to -1)*	*Avg Gains*	0.80	0.32	0	0	0	0.76
	*N*	2687	237	0	0	0	2924
*[-1 to -0.5)*	*Avg Gains*	0.54	0.44	0.21	0	0	0.45
	*N*	658	1670	176	0	0	2504
*[-0.5 to 0)*	*Avg Gains*	0.42	0.47	0.34	0.15	0	0.37
	*N*	279	1171	1822	416	0	3688
*[0 to 0.5)*	*Avg Gains*	0	0.39	0.36	0.32	0.05	0.31
	*N*	0	546	1265	1883	488	4182
*[0.5–1)*	*Avg Gains*	0	0	0.32	0.28	0.22	0.26
	*N*	0	0	358	1248	1425	3031
*[1–5)*	*Avg Gains*	0	0	0	0.14	0.12	0.12
	*N*	0	0	0	77	1708	1785

Notes: This table shows the forward yearly test score gains (i.e. $y_{it+1}-y_{it}$) for students given their test score at time *t* (rows) and their test score performance quintile at time *t* (columns). The number of observations in each group is also shown. Specifically, we first score children on a common linked scale as described in the text. Then, we construct *within* grade quintiles, so that children with the same score may be in different quintiles depending on what grade they were in. For instance, children with very low scores [-5 to -1) are mostly in the bottom quintile (Quintile 1) with less than 10 % in the 2nd Quintile. Children with scores between [0 to 0.5) are distributed across Quintile 2 and 5. If, for instance, this score was observed in Grade 3, they would likely be in Quintile 4 or 5, but if this score was observed in Grade 5, they may be in Quintile 2 or 3. We then show the average gain in test scores in the following year for each test-score interval and quintile. For instance, the 546 children who scored between [0 to 0.5) but were in the 2nd quintile gained an average of 0.39SD, but the 488 children who scored between [0 to 0.5) but were in the top quintile gained 0.

**Table A8:** Correlates variables definitions.

Variable	Variable Name	Definition
Asset Index (School-level data)	sc_pca_4years	Predicted first Principal Component index scaled to have mean 0 across all years. It uses assets collected from a random sample of children at each school. The PCA uses assets data from 2003−06.
Assets Index (HH-level data)	hh_pca_4years	Predicted first Principal Component index scaled to have mean 0 across all years. It uses assets collected at the household. The PCA uses assets data from 2003−06.

Mother Education Groups (School-level data)	sc_mother_educ	Mean of available parental education groups in 2003−06 at the school-level. Missings are ignored to estimate the mean, and results are rounded to the closest unit.
		Parental groups follow this definition:
		1 = No Education
		2 = Less than Primary (less than Grade 5 - did not pass Grade 5 exams)
Father Education Groups (School-level data)	sc_father_educ	3 = Greater than Primary to Higher Secondary (greater or equal than Grade 5 to less or equal than Grade 12)
		4 = Higher Secondary or higher (greater than Grade 12)

Mother Education Groups (HH-level data)	hh_mother_educ	First, years of parental education is defined as the average across all observed years (ignoring missings) of the highest grade of formal schooling completed by each parent. When above 12, the following assumptions are made: (i) BA/BSC/B.Ed = 15; and (ii) MA/MSC/M.Ed/MBA = 17.
Father Education Groups (HH-level data)	hh_father_educ	Then, these parental education groups are constructed from years of parental education using the following definition:
		1 = No Education
		2 = Less than Primary (less than Grade 5 - did not pass Grade 5 exams)
		3 = Greater than Primary to Higher Secondary (greater or equal than Grade 5 to less or equal than Grade 12)
		4 = Higher Secondary or higher (greater than Grade 12)

Test Items	eng_item*	Each variable assumes that unanswered questions that were asked are marked as wrong. Only typos (i.e. values different than 0 for incorrect and 1 for correct) are set to missing if the question was asked.
	math_item*	
	urdu_item*	Variables take the value of missing if question is NOT asked in a given year.

## Appendix A ATTRITION

### Exploration of Attrition Patterns in the Data

To understand whether the patterns of number of students observed for 1, 2, 3, and 4 years in Table 1 would occur under random attrition, we carried out a number of simulations for the 3-year data. These are shown in Appendix Table A2, with detailed explanation of the exercise in the notes to the table. We present data from three years because, in the fourth year, some of the missing test scores could be because of higher dropouts. In these simulations, we assume that there is some degree of absence in each year. We also assume that between 3% and 7% of children are misclassified (i.e. classified as a new individual in the panel when, in fact, they had been observed before) in every year.

Interestingly, it is quite hard to replicate observed patterns across the entire distribution with random attrition. The fraction of children observed for 3 years is consistent with absence rates of 15 % and misclassification between 3 % and 5 %, but in this case, we should have seen fewer children with only one observation. Alternately, parameters that correctly predict the number of children we see only once under-predict the number of children with 3 years of data. Since we never observe test score gains for these “sporadic” children, they are effectively eliminated from our sample—but remain an important group of children that requires further investigation.^30^

Our working assumption remains that the data reflect 15 % absenteeism, which may be selective, as well as 3 %–5 % misclassification in each year, which is likely to be random. To understand learning trajectories, we can treat the data as a cross-section in every year, discarding the panel aspect of the data, and these trajectories will be biased to the extent that absence and test score gains are correlated in these data.

### Using Intensively Tracked Sample to Characterize Bias

Suppose that in addition to the school sample, we also have an “intensively” tracked sample where a greater fraction of children are observed for all four years. Label the child’s “true” knowledge level in a given year as $x_{it}$, where *i* is the child and *t* the year so that a child’s true learning gain is $x_{it}-x_{i,t-1}=\delta_{i}$. We observe test scores, which are given by $y_{it}=x_{it}+\nu_{it}$, where $\nu_{it}$ is a random idiosyncratic error. Then, if we observe all students’ test scores, the average learning gains are an unbiased estimate of average learning $E(\delta_{i})$. However, in a given year, we only observe an individual if $A_{i}+\varepsilon_{it}>z$, where $A_{i}$ is an individual-level, unobserved characteristic that may be correlated with $\delta_{i}$, and $\varepsilon_{it}$ is an individual-year idiosyncratic shock. Then, we can write the probability of being observed as $p(A_{i})$. The estimate of learning gains is then $\hat{\delta }=\frac{E(p\left(A_{i}\right)\delta_{i})}{E(p\left(A_{i})\right)}=\frac{Cov\left(p\left(A_{i}\right),\delta_{i}\right)+E\left(p\left(A_{i}\right)\right)E(\delta_{i})}{E(p\left(A_{i}\right))}=\frac{Cov\left(p\left(A_{i}\right),\delta_{i}\right)}{E(p\left(A_{i}\right))}+E(\delta_{i})$. So, the bias in the estimate is $\frac{Cov\left(p\left(A_{i}\right),\delta_{i}\right)}{E(p\left(A_{i}\right))}$, and there is no bias due to attrition if attrition is not correlated with $\delta_{i}$ (that is, $Cov\left(p\left(A_{i}\right),\delta_{i}\right)=0$). Now we can consider two samples where one is more intensively and one is less intensively tracked. For the more intensively tracked sample, we can say that a student is observed if $A_{i}+\varepsilon_{it}>z-\text{Δ}z$, which can be written as $A_{i}+\text{Δ}z+\;\varepsilon_{it}>z$. Then the difference in the estimates from the two samples is $\frac{Cov\left(p\left(A_{i}+\text{Δ}z\right),\delta_{i}\right)}{E(p\left(A_{i}+\text{Δ}z\right))}$ - $\frac{Cov\left(p\left(A_{i}\right),\delta_{i}\right)}{E(p\left(A_{i}\right))}$. Note that this value is equal to 0 if $Cov\left(p\left(A_{i}+\text{Δ}z\right),\delta_{i}\right)=Cov\left(p\left(A_{i}\right),\delta_{i}\right)=0$ (that is, if there is no selection bias) but otherwise it is not equal to 0. Additionally, if we are willing to assume $v\left(p\left(A_{i}+\text{Δ}z\right),\delta_{i}\right)\approx Cov\left(p\left(A_{i}\right),\delta_{i}\right)$, we note that the difference between the two estimates will be strictly increasing in the size of the bias.

### Stability of Results Across Samples with Different Degrees of Attrition

Appendix Table A4 compares learning for 3 different samples —a balanced sample of 6038 children who were present in every year, the unbalanced samples of children present in different years, and the household sample where the balanced proportion is higher. Again, test score gains are very similar across all three samples. There are statistically significant differences between the unbalanced/balanced panels when comparing against the household panel but only when learning includes year 4, which is likely caused by the inclusion of testing dropouts at home and the fact that the *balanced* panel itself is a (slightly) selected group of children. However, these differences are small in magnitude (less than 0.06 SD) and suggest that absences can be treated as equivalent to “missing at random” (with respect to learning gains) for our computations.

## Appendix B COMPARISON OF GAINS AND VALUE-ADDED SPECIFICATIONS

Instead of a gains specification, Muralidharan, Singh and Ganimian (2019) use a value-added specification to test whether test scores converge or diverge in their sample. They run the regression $y_{it}=\;\beta_{0}\;+\lambda y_{it-1}+\sum_{q}\delta_{q}Q^{q}+\;\varepsilon_{it}$, where *q* sums over the quartiles of lagged test scores within a grade, $Q^{q}$ is an indicator variable equal to 1 if a student is in quartile *q*, and as in the main text $y_{it}$ is the test score for a student *i* in a year *t*. Identification is achieved because $y_{it-1}$ is computed across grades, while quartiles are grade specific. They show that the estimated coefficient on the lowest quartile is approximately 0. When children at the same score are in a lower quartile because they are in a higher grade, their gains are lower. This specification relies on the assumption that λ is a global parameter with constant effects across quartiles and grades. When we replicate their specification in Appendix Table A6, we find evidence of divergence, in line with their findings in India. We note, however, that assuming a constant value of the persistence parameter λ is itself problematic. If we instead use a non-parametric table, where we simply examine grades by test-score and quartiles (again, quartiles for the same test score are different because children are in different grades), we find similar patterns of convergence as we have documented before (see Appendix Table A7).

## Footnotes

2 Data for Florida were generously made available to us by David Figlio at Northwestern University.

3 LEAPS only tracked dropouts between Grades 5 and 6 and not in lower grades. Therefore, our results are limited to dropouts between these grades. The similarity of test score gains does not imply that rates of learning are identical, as these samples were administered different tests.

4 These studies test a sample of children in treated and control areas, regardless of enrollment status. If children who are incentivized to remain in school also gain test scores, the studies should have found that children in treated areas have higher test scores than those in control areas.

5 Furthermore, completed schooling at age 22 is strongly correlated with test scores at age 12 in both the LEAPS and the Young Lives samples (Das et al., 2020).

6 Since (a) schools open and close, and (b) in 2006, some children, who had moved on to middle school (Grade 6), were studying in schools outside the village, the total number of schools and villages is higher for the 4 years of testing.

7 The residual 1% corresponds to NGOs/community schools.

8 Some children, who were held-back or double-promoted, were tested in their new grades.

9 In practice, since we were following all schools and children in the relevant grade, we were successful in tracking children even when they moved in most cases. However, some children had similar names or used variants of the same name (Mohammed Abdul Karim may be in School A in year 2003 but then may move to School B in year 2004 and be registered as Abdul Karim) leading to inevitable uncertainty in these limited cases.

10 Maximum likelihood estimates (MLE) of the underlying latent trait θ are used throughout this paper.

11 Although other cohorts were tracked over the years, IRT parameters are estimated using the 4 rounds of LEAPS test score data collected for the cohort of children in Grade 3 in 2003.

12 The model assumes a true distribution of θ with mean 0 and a SD of 1 in the presence of infinite data. In practice, the finite nature of our sample yields a distribution very close to the desired one but with minor deviations.

13 A specific example of a question with differential item functioning in Pakistan is asking children to translate numbers into words (for instance, 22 is “Twenty-Two”). In our pilot, we noticed that children in schools taught predominantly in Urdu, as opposed to English, found these questions to be harder. The reason is that in Urdu, *every* number between 1 and 100 is different, while in English, once you know the numbers 1 through 10 in words as well as 20 (twenty), 30 (thirty), etc., numbers like 44 are easier to translate into words. Therefore, conditional on knowledge, children taught in Urdu find these questions harder than those taught in English.

14 An important caveat is that children who are only observed once are never included in our calculations of learning gains since they do not have lagged test scores. We further discuss this group and the implications for our estimates in Appendix A.

15 Test scores in 2003 were -0.56 SD in the household sample compared to -0.55 SD in the school sample and are therefore also statistically indistinguishable.

16 These gains are not systematically biased due to the unbalanced nature of our sample. We find very similar gains whether we look at the unbalanced or balanced school sample or the household sample where the fraction of children with test scores in every year is much higher.

17 Muralidharan, Singh and Ganimian (2019) report results from middle school. We discuss their results below.

18 Using the notation above and denoting the lower and upper boundaries of a quintile as z^l^ and z^h^, we are estimating $E\left(y_{it}-y_{it-1}\right|z^{l}<x_{it-2}+\nu_{it-2}<z^{h})=E\left(x_{it}-x_{it-1}\right|z^{l}<x_{it-2}+\nu_{it-2}<z^{h}),$ while for the gains for the “true” ability quintile are $E\left(x_{it}-x_{it-1}\right|z^{l}<x_{it-2}<z^{h})$.

19 We construct the asset index using a principle component approach. Appendix Fig. A5 shows that an alternate IRT approach yields similar results with a correlation of 0.96 between the two measures.

20 For instance, 1 SD can amount to the difference between “knowing” how to add and subtract or the difference between “knowing” how to count and calculus in a particular math test. Unless the test satisfies the assumptions of a Rasch model, any monotonic transformation of the score is also a theoretically possible and alternate measure of knowledge on the test (Lord, 1975). Bond and Lang (2013) show that the differences in test score gains between Blacks and Whites depends on what transformation is chosen—both convergence and divergence can be rationalized using different transformations.

21 Since purposely searching for transformations that maximize and minimize the desired learning gains gaps often yields wide bounds, we also discard very unlikely transformations, specifically those with skewness outside [-2,2] and/or kurtosis outside [0,10], as suggested by Ho and Yu (2015) in their assessment of likely test score distribution characteristics.

22 Similar numbers from multiple cross-sections in low-income countries contribute to the idea of a learning crisis in these settings.

23 This estimate comes from an additional long-term follow-up LEAPS round that is not used in this paper.

24 This finding continues to be debated with regard to race, sex, and socio-economic status in the United States but appears to hold in multiple datasets (see for instance, Carneiro and Heckman 2000).

25 Consider two children, one who scores 1 in year *t-1* and 2 in year *t* and another who scores 2 in year *t-1* and 3 in year *t*. Both children have equal test score gains. But when λ=.7, the first child gains 1.3 and the second gains 1.6.

26 Andrabi et al. (2011) also showed that naive estimates of $\gamma_{1}$ (like in the value-added specification above) are biased downwards due to measurement error and biased upwards due to omitted variables (a child with higher $y_{it-1}$ may have unobserved ability, which will also directly affect $y_{it}$). Incorrectly estimating λ can greatly affect the estimated $\gamma_{1}$, depending on the covariance ($y_{it-1}\;,\;X_{it}$). In the LEAPS data these two biases cancel each other out—the estimate of λ, after correcting for measurement error and omitted variable bias, is similar to what we would obtain in the specification$\;y_{it}\;=\;\gamma_{0}+\gamma_{1}X_{it}+\lambda y_{it-1}+\;\eta_{it}$.

27 Both guessing and concavity are already accounted for in the IRT procedure. The guessing parameter is estimated from the data, rather than stipulated as the inverse of the number of options because sometimes children leave the question blank and sometimes there may be a ‘trick’ that leads children to a wrong answer with higher probability. Cardinality is addressed through the difficulty and discrimination parameters, assuming that the stringent assumptions required are valid for this test; it is also partially addressed through the Bond-Lang procedure discussed previously. Finally, vertically linked test scores are critical for the interpretation of β as the lack of persistence in learning levels. If instead we “standardized” test scores to have a variance of 1 in every year, then mechanically, the persistence coefficient is a function of the variance of the error term, even if learning is strictly increasing. This is because $var\left(y_{it}\right)=\beta^{2}var\left(y_{i,t-1}\right)+var(\varepsilon_{it})$. Therefore, if $var\left(y_{it}\right)=var\left(y_{it-1}\right)=1$, $\beta^{2}=1-var(\varepsilon_{it}\;)<1$ unless $var\left(\varepsilon_{it}\right)=0$. Intuitively, this is because when scores are standardized within-years, the score is only capturing a student’s ranking in the distribution, rather than her accumulated knowledge.

28 For instance, the guessing parameter, *c,* for MCQ English Items 29, 30, 45 and 46 is between 0.14 and 0.16, while it is less than 0.002 for any of the non-MCQ English Items. This difference is generally true for most other English, Math, and Urdu items, but with smaller absolute differences since estimated guessing parameters are relatively low for several MCQ questions as well.

29 See Department of Basic Education and the University of the Witwatersrand (2020)
